# Programmable DNA‐Based Molecular Neural Network Biocomputing Circuits for Solving Partial Differential Equations

**DOI:** 10.1002/advs.202507060

**Published:** 2025-06-25

**Authors:** Yijun Xiao, Alfonso Rodríguez‐Patón, Jianmin Wang, Pan Zheng, Tongmao Ma, Tao Song

**Affiliations:** ^1^ Qingdao Institute of Software, College of Computer Science and Technology China University of Petroleum Qingdao 266580 China; ^2^ Departamento de Inteligencia Artificial, ETSIINF Universidad Politécnica de Madrid Madrid 28040 Spain; ^3^ Department of Integrative Biotechnology Yonsei University Incheon 03722 Korea (the Republic of); ^4^ Information Systems Group University of Canterbury Christchurch 8041 New Zealand

**Keywords:** chemical reaction networks (CRNs), DNA computing, DNA strand displacement reactions, neural networks circuits, partial differential equations

## Abstract

Partial differential equations, essential for modeling dynamic systems, persistently confront computational complexity bottlenecks in high‐dimensional problems, yet DNA‐based parallel computing architectures, leveraging their discrete mathematics merits, provide transformative potential by harnessing inherent molecular parallelism. This research introduces an augmented matrix‐based DNA molecular neural network to achieve molecular‐level solving of biological Brusselator PDEs. Two crucial innovations address existing technological constraints: (i) an augmented matrix‐based error‐feedback DNA molecular neural network, enabling multidimensional parameter integration through DNA strand displacement cascades and iterative weight optimization; (ii) incorporating membrane diffusion theory with division operation principles into DNA circuits to develop partial differential calculation modules. Simulation results demonstrate that the augmented matrix‐based DNA neural network efficiently and accurately learns target functions; integrating the proposed partial derivative computation strategy, this architecture solves the biological Brusselator PDE numerically with errors below 0.02 within 12,500 s. This work establishes a novel intelligent non‐silicon‐based computational framework, providing theoretical foundations and potential implementation paradigms for future bio‐inspired computing and unconventional computing devices in life science research.

## Graphical abstract

This research introduces an augmented matrix‐based DNA molecular neural network to achieve molecular‐level solving of biological Brusselator PDEs. Crucial innovations include: (i) an augmented matrix‐based DNA molecular neural network, enabling multidimensional parameter integration through DNA strand displacement cascades and iterative weight optimization; (ii) incorporating membrane diffusion theory with division operation principles into DNA circuits to develop partial differential calculation modules.

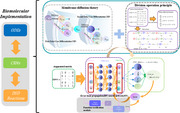

## 1 Introduction

Partial Differential Equations are the core mathematical tools used to characterize the dynamic behavior of physical, biological, and engineering systems. Efficient solutions to PDEs are essential for critical areas such as climate modeling and drug diffusion simulation.^[^ ^1^, ^2^, ^3^ ^]^ However, traditional numerical methods, such as the finite element method (FEM) and the finite difference method (FDM), encounter fundamental challenges in solving high‐dimensional nonlinear PDEs. The computational complexity and cost increase exponentially as dimensionality grows, known as the curse of dimensionality. In recent years, biomolecular systems have garnered significant attention due to their natural parallelism and energy‐efficient properties,^[^ ^4^, ^5^, ^6^, ^7^, ^8^ ^]^ providing new avenues for nontraditional computational frameworks based on biomolecular systems. The programmable and parallel computational capabilities of DNA molecules^[^ ^9^ ^]^ through strand displacement reactions provides a new idea for continuous‐space differential arithmetic‐through the gradient distribution of molecular concentration and the dynamic reaction network, it is expected to map directly the differential operators of PDEs, circumventing the loss of accuracy caused by traditional discretization. This bio‐physical fusion computing architecture provides the possibility of breaking through the traditional computational complexity boundaries for the development of a new generation of PDE solvers.

Solving high‐dimensional PDE remains a formidable challenge in computational science, where conventional numerical methods suffer from the curse of dimensionality that compromises both accuracy and efficiency. Recent advances in deep neural networks leverage nonlinear activation functions and continuous function approximation to adaptively capture high‐dimensional and nonlinear features, eliminating the requirement for precise analytical models or mesh generation. However, conventional silicon‐based neural architectures face inherent limitations in energy efficiency and physical interconnect density imposed by von Neumann architectures, hindering their capability for massively parallel computation of ultra‐large‐scale PDE systems.^[^ ^10^, ^11^ ^]^

Non‐silicon‐based DNA neural networks provide a transformative paradigm to resolve this conflict. Molecular reactions in DNA computation exhibit extraordinary parallelism (“epi‐bit” parallel DNA molecular data storage^[^ ^12^ ^]^) and ultra‐low energy consumption (DNA‐based programmable gate arrays, DPGA^[^ ^13^ ^]^). While neural networks demonstrate superior approximation capacity for PDE solutions, their electronic implementations encounter fundamental bottlenecks in power dissipation and scalability. The chemical implementation of neural networks through programmable DNA reactions offers a disruptive computational framework that inherently bypasses these limitations through massive molecular parallelism. Cherry and Qian^[^ ^14^ ^]^ DNA extends the molecular pattern recognition capabilities of DNA neural networks by constructing a winner‐takes‐all neural network strategy to recognize nine molecular patterns. Zou et al.^[^ ^15^, ^16^ ^]^ designed a nonlinear neural network based on the DNA strand replacement reaction, which was then utilized to perform standard quadratic function learning. In addition, they tested the robustness of the nonlinear neural network to detection of DNA strand concentration, the strand replacement reaction rate, and noise. The DNA molecular neural network system demonstrates adaptive behavior and supervised learning capabilities, demonstrating the promise of DNA displacement reaction circuits for artificial intelligence applications. Zou^[^ ^17^ ^]^ also proposed a novel activation function that was embedded in a DNA nonlinear neural network to achieve the fitting and prediction of specific nonlinear functions. The function can be realized by enzyme‐free DNA hybridization reactions and has good nesting properties, allowing the construction of complete circuits in cascade with other DNA reactions. Xiong et al.^[^ ^18^ ^]^ addressed the key scientific challenges of developing an ultra‐large‐scale DNA molecular response network by achieving independent regulation of the signal transmission function and the weight‐giving function for weight sharing based on modularity of the functions of the weight‐regulating region and the recognition region, with the molecular switch gate architecture as the basic circuit component. A large‐scale DNA convolutional neural network (ConvNet) has been constructed by cascading several modular molecular computing units with synthetic DNA regulatory circuits, which is capable of recognizing and classifying 32 classes of 144‐bit molecular information profiles. The developed DNA neural network has robust molecular mapping information processing capacity that is predicted to be utilized in intelligent biosensing and other fields.

DNA's programmability, adaptability, and biocompatibility^[^ ^19^ ^]^ make it an ideal candidate for integrating computational and control applications in synthetic biology. DNA strand displacement (DSD) reaction‐driven architectures enable the development of biochemical circuits from DNA strand sequences, allowing for digital computing and analog simulation.^[^ ^20^, ^21^, ^22^, ^23^, ^24^, ^25^ ^]^ Previous research has demonstrated the promise of DNA circuits for pattern classification and optimization issues,^[^ ^26^, ^27^, ^28^ ^]^ but the implementation of continuous mathematical operations (such as partial derivative computations) remains a challenge.

This research provides a methodology for solving partial differential equations using DNA molecular neural network based on an augmentation matrix. Its main contributions include: (i) constructing a DSD reaction‐driven augmentation matrix‐based DNA molecular neural network, mapping the weights to the DNA strand concentration by introducing the augmentation matrix encoding strategy, and realizing multi‐parameter combination output by utilizing parallelism of DNA toehold mediated displacement reaction to realize the output of multi‐parameter combination. (ii) deeply integrating the membrane diffusion theory and division operation principles into DNA circuits, proposing a scheme for calculating partial derivatives using DSD reactions, and then simulating the partial derivatives process, which breaks through the theoretical limitations of molecular systems in continuous mathematical operations; (iii) constructing the first DNA molecular neural network architecture to support the solution of partial derivative equations, which achieves adaptive adjustment of the weight parameters through an error‐feedback mechanism. This research develops new partial differential computational strategies for biomolecular computers while also providing a non‐silicon based computational device, facilitating a significant transition from discrete logic to continuous mathematics in biocomputing.

## 2 Methodology

### 2.1 CRNs‐Based Multi‐Combinatorial Parameter Parallel Output Neural Networks

#### 2.1.1 CRNs‐Based Error Back Propagation Neural Network

**Figure 1:**
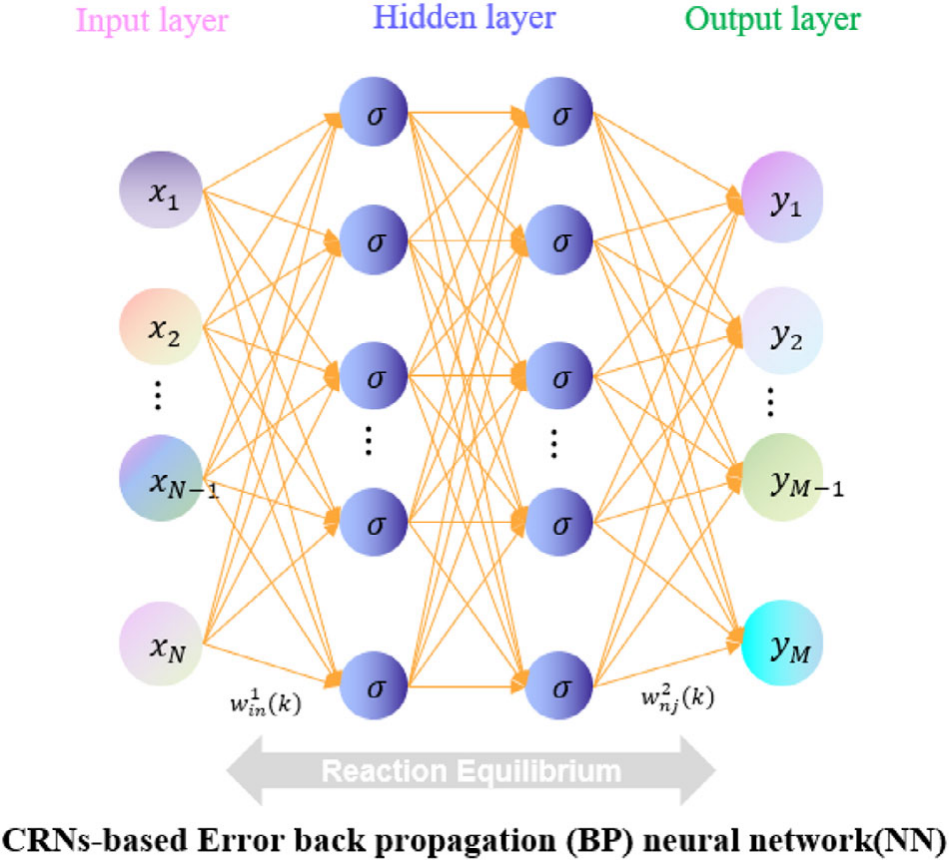
CRNs‐based error back propagation neural network.

Assuming N input parameters of a neural network, that is *X*(*k*) = [*x*_1_(*k*), *x*_2_(*k*), …, *x*_*N*_(*k*)]^*T*^, weights ${\left(W_{i}^{1}\right)}^{T}=\left[w_{i0}^{1},w_{i1}^{1},\text{…},w_{iL}^{1}\right]$ are utilized to connect the i‐th unit of the input layer to multiple units of the hidden layer, where *i* = 1, 2, 3, …, *N*, (*L* + 1) denotes the number of units in the hidden layer; weights ${\left(W_{n}^{2}\right)}^{T}=\left[w_{n0}^{2},w_{n1}^{2},\text{…},w_{nM}^{2}\right]$ are used to connect the n‐th unit of the hidden layer with multiple units of the output layer; (*M* + 1) denotes the number of units in the output layer; $\theta_{n}^{1}$ and $\theta_{j}^{2}$ are the thresholds of the input and hidden layers, respectively. The outputs of the hidden and output layers are denoted by $y_{n}^{1}\left(k\right)$ and *y*_*j*_(*k*), respectively, where *n* = 1, 2, 3, …, *L* and *j* = 1, 2, 3, …, *M*. The entire procedure depicted in **Figure** **1** can be characterized as:

(1) $$\left\{\begin{array}{cc} s_{n}^{1}\left(k\right) & =\sum_{i=1}^{N}w_{in}^{1}x_{i}\left(k\right)-\theta_{n}^{1}\\ y_{n}^{1}\left(k\right) & =\psi (s_{n}^{1}\left(k\right))\\ s_{j}^{2}\left(k\right) & =\sum_{n=1}^{L}w_{nj}^{2}y_{n}\left(k\right)-\theta_{j}^{2}\\ y_{j}\left(k\right) & =\psi (s_{j}^{2}\left(k\right)) \end{array}\right.$$

where ψ(*) represents the activation function.

**CRNs‐based input layer weighted sum (including threshold) calculation**:

(2) $$\left\{\begin{array}{cc} & X_{i}+W_{in}+I_{n}\overset{k_{1}}{\to }X_{i}+2I_{n}\\ & I_{n}+S_{n}\overset{k_{1}}{\to }S_{n}\\ & I_{n}+\theta_{n}\overset{k_{1}}{\to }⌀ \end{array}\right.\Rightarrow \left\{\begin{array}{cc} & X_{1}+W_{11}+I_{1}\overset{k_{1}}{\to }X_{1}+2I_{1}\\ & \;\vdots \\ & X_{N}+W_{N1}+I_{1}\overset{k_{1}}{\to }X_{N}+2I_{1}\\ & \;\vdots \\ & X_{1}+W_{1L}+I_{L}\overset{k_{1}}{\to }X_{1}+2I_{L}\\ & \;\vdots \\ & X_{N}+W_{NL}+I_{L}\overset{k_{1}}{\to }X_{N}+2I_{L}\\ & I_{1}+S_{1}\overset{k_{1}}{\to }S_{1}\\ & \;\vdots \\ & I_{L}+S_{L}\overset{k_{1}}{\to }S_{L}\\ & I_{1}+\theta_{1}\overset{k_{1}}{\to }⌀\\ & \;\vdots \\ & I_{L}+\theta_{L}\overset{k_{1}}{\to }⌀ \end{array}\right.$$

**CRNs‐based hidden layer activation function**: Taking the activation function ψ(*) = (*)^2^ as an example, the corresponding CRNs can be described as follows:

(3)

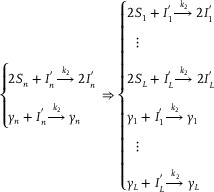




**CRNs‐based output layer activation function**: Taking the activation function ψ(*) = (*)^2^ as an example, the corresponding CRNs can be given:
(4)

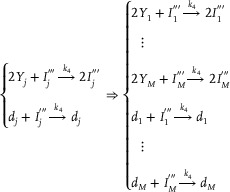




**CRNs‐based output layer weighted sum (including threshold) calculation**:
(5)

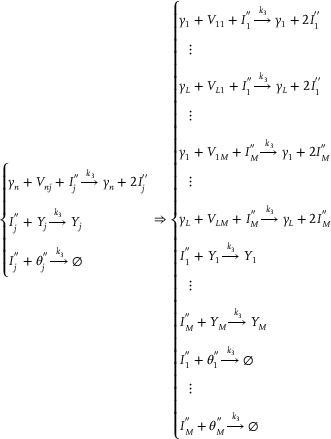




**CRNs‐based weight update mechanism**: The weight update of the back‐propagation neural network can be achieved utilizing the gradient descent algorithm:

(6) $$\left\{\begin{array}{cc} & w_{in}^{1}\left(k+1\right)=w_{in}^{1}\left(k\right)+\alpha \Delta w_{in}^{1}\left(k\right)\\ & w_{nj}^{2}\left(k+1\right)=w_{nj}^{2}\left(k\right)+\alpha \Delta w_{nj}^{2}\left(k\right) \end{array}\right.$$

where α(0 < α < 1) is the learning step size, $\Delta w_{in}^{1}\left(k\right)=\delta_{n}^{1}\left(k\right)x_{i}\left(k\right)$, and $\Delta w_{nj}^{2}\left(k\right)=\delta_{j}^{2}\left(k\right)y_{n}^{1}\left(k\right)$. $\delta_{n}^{1}$ and $\delta_{j}^{2}$ are the back‐propagated errors.

Remark 1 The relationship between the neural network weight update and the loss function Loss can be indicated as follows:

(7) $$\;w_{i}^{\text{new}\;}=w_{i}^{\text{old}\;}-\eta \frac{\partial (\;\text{Loss}\;)}{\partial w}\;$$

When substituting Equation (7) into Equation (6), the expression can be reformulated as:

(8) $$\left\{\begin{array}{cc} & w_{in}^{1}\left(k+1\right)=w_{in}^{1}\left(k\right)+\alpha \Delta w_{in}^{1}\left(k\right)\\ & \;=\;w_{in}^{1}\left(k\right)+\alpha \frac{\partial (Loss)}{\partial w_{in}}\\ & w_{nj}^{2}\left(k+1\right)=w_{nj}^{2}\left(k\right)+\alpha \Delta w_{nj}^{2}\left(k\right)\\ & \;=\;w_{nj}^{2}\left(k\right)+\alpha \frac{\partial (Loss)}{\partial w_{nj}} \end{array}\right.$$

CRNs‐based weight update mechanism can be described as:

(9) $$\begin{array}{cc} & W_{in}+La_{in}\underset{k_{6}}{\overset{k_{5}}{⇄}}Lb_{in}\\ & V_{nj}+Lc_{in}\underset{k_{8}}{\overset{k_{7}}{⇄}}Ld_{in}\\ & S_{n}+Le_{n}\underset{k_{10}}{\overset{k_{9}}{⇄}}Lf_{n}\\ & y_{n}+Lg_{n}\underset{k_{12}}{\overset{k_{11}}{⇄}}Lh_{n}\\ & Y_{j}+Lm_{j}\underset{k_{14}}{\overset{k_{13}}{⇄}}Ln_{j} \end{array}$$

where *k*_*m*_(*m* = 5, 7, 9, 11, 13) is the forward reaction rate, and *k*_*n*_(*n* = 6, 8, 10, 12, 14) is the reverse reaction rate. The initial concentrations of [*W*_*in*_, *La*_*in*_, *Lb*_*in*_], [*V*_*nj*_, *Lc*_*in*_, *Ld*_*in*_], [*S*_*n*_, *Le*_*n*_, *Lf*_*n*_], [*y*_*n*_, *Lg*_*n*_, *Lh*_*n*_], and [*Y*_*j*_, *Lm*_*j*_, *Ln*_*j*_] satisfy the following conditions:

(10) $$\begin{array}{cc} & {\left[W_{in}\right]}_{0}\ll {\left[Lb_{in}\right]}_{0},{\left[La_{in}\right]}_{0}\ll {\left[Lb_{in}\right]}_{0}\\ & {\left[V_{nj}\right]}_{0}\ll {\left[Ld_{in}\right]}_{0},{\left[Lc_{in}\right]}_{0}\ll {\left[Ld_{in}\right]}_{0}\\ & {\left[S_{n}\right]}_{0}\ll {\left[Lf_{n}\right]}_{0},{\left[Le_{n}\right]}_{0}\ll {\left[Lf_{n}\right]}_{0}\\ & {\left[y_{n}\right]}_{0}\ll {\left[Lh_{n}\right]}_{0},{\left[Lg_{n}\right]}_{0}\ll {\left[Lh_{n}\right]}_{0}\\ & {\left[Y_{j}\right]}_{0}\ll {\left[Ln_{j}\right]}_{0},{\left[Lm_{j}\right]}_{0}\ll {\left[Ln_{j}\right]}_{0} \end{array}$$

According to Reactions (2)‐(5), the ordinary differential equations (ODEs) of substances *I*_*n*_, 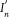, 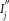, and 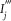 can be written as:

(11)

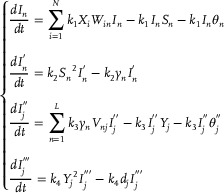

When the concentrations of substances *I*_*n*_, 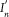, 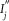 and 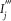 achieve steady‐state equilibrium, that is $\frac{dI_{n}}{dt}=0$, 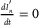, 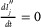, and 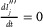, the following results can be obtained:

(12)

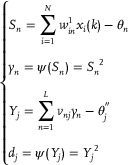




#### 2.1.2 Realizing Multi‐Combination Variable Output Using Augmented Matrix Neural Network

**Figure 2:**
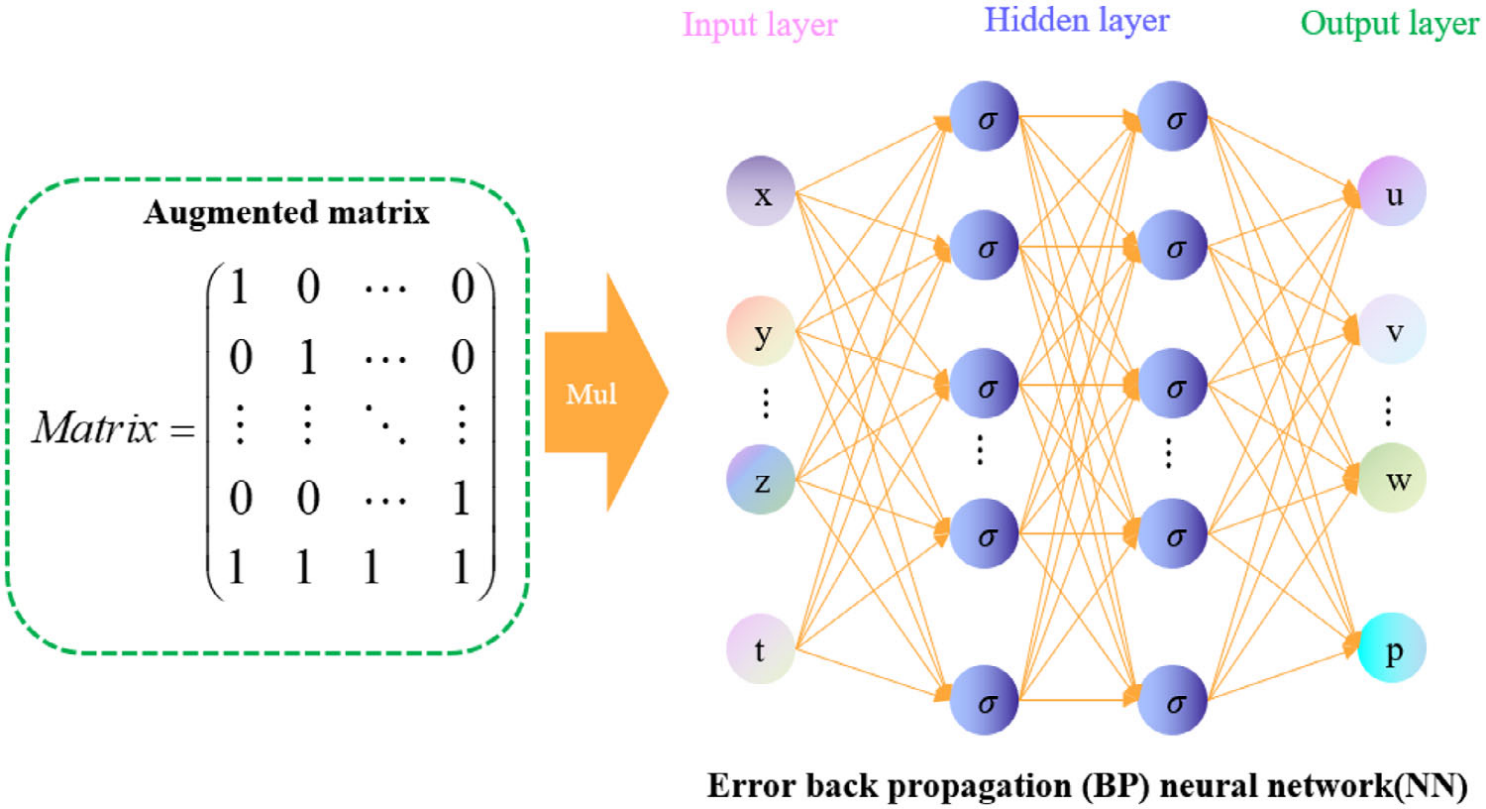
Error back propagation neural network architecture diagram based on augmented matrix.

**Figure** **2** depicts the structure of a backpropagation neural network utilizing the augmented matrix. By using the augmented matrix represented in the left sub‐figure, the neural network's input parameter setting could be indirectly controlled via the multiplication operation, allowing the neural network to regulate its output with regard to numerous combination parameters.

The functional relationship between the input and output layers of the right subgraph has been rigorously demonstrated in Equation (12). The augmented matrix parameter is applied to the first sub‐equation, which is equivalent to introducing an additional parameter ξ into $\sum_{i=1}^{N}w_{in}^{1}x_{i}\left(k\right)$, denoted as $\sum_{i=1}^{N}\xi w_{in}^{1}x_{i}\left(k\right)$, where ξ is assigned a value of either 0 or 1. Noted that the augmented matrix in Figure 2 specifically refers to an augmented identity matrix, constructed by appending an additional row of unity elements to the conventional identity matrix. This particular matrix configuration constitutes a critical implementation aspect of the proposed methodology.

Assuming that the augmented matrix is the augmented identity matrix, the following results can be obtained:

(13) $$\begin{array}{cc} X(k)•\;\text{Matrix}\; & ={\left[x_{1}\left(k\right),x_{2}\left(k\right),\text{…},x_{N}\left(k\right)\right]}^{T}•\left(\begin{array}{cccc} 1 & 0 & \cdots & 0\\ 0 & 1 & \cdots & 0\\ \vdots & \vdots & \ddots & \vdots \\ 0 & 0 & \cdots & 1\\ 1 & 1 & \cdots & 1 \end{array}\right)\\ & =\left(\begin{array}{c} x_{1}\left(k\right)\\ x_{2}\left(k\right)\\ \vdots \\ x_{N-1}\left(k\right)\\ x_{N}\left(k\right) \end{array}\right)\left(\begin{array}{cccc} 1 & 0 & \cdots & 0\\ 0 & 1 & \cdots & 0\\ \vdots & \vdots & \ddots & \vdots \\ 0 & 0 & \cdots & 1\\ 1 & 1 & \cdots & 1 \end{array}\right)\\ & =\left(\begin{array}{cccc} x_{1}\left(k\right) & 0 & \cdots & 0\\ 0 & x_{2}\left(k\right) & \cdots & 0\\ \vdots & \vdots & \ddots & \vdots \\ 0 & 0 & \cdots & x_{N}\left(k\right)\\ x_{1}\left(k\right) & x_{2}\left(k\right) & \cdots & x_{N}\left(k\right) \end{array}\right) \end{array}$$

Equation (12) describes the relationship between each neural network output *d*_*j*_ and the input parameter *x*_*i*_ as follows: 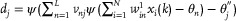. By combining Equations (12) and (13), we obtain the following neural network output matrix.

(14)

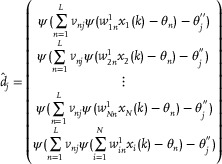




### 2.2 Principle of CRNs‐Based Partial Derivative Calculation

Further analysis of Equation (14) yields:

(15)

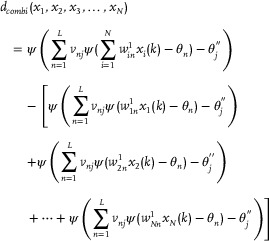

In Equation (15), *d*_*combi*_(*x*_1_, *x*_2_, *x*_3_, …, *x*_*N*_) represents the sum of the combined terms of at least two variables in the variable output *x*_1_, *x*_2_, *x*_3_, …, *x*_*N*_ by the neural network. If *d*_*combi*_(*x*_1_, *x*_2_, *x*_3_, …, *x*_*N*_) = 0, it means that the function learned by the neural network does not contain any composite terms; conversely, *d*_*combi*_(*x*_1_, *x*_2_, *x*_3_, …, *x*_*N*_) ≠ 0 means that the function learned by the neural network has composite terms. To determine whether there are composite terms, the partial derivative calculation of the input variable *x*_1_, *x*_2_, *x*_3_, …, *x*_*N*_ is taken into account.

According to the partial derivative calculation principle, the corresponding partial derivative is calculated for the neural network output ${\hat{d}}_{j}$:

(16) $$\begin{array}{ccc} & & \left\{\begin{array}{cc} & \frac{\partial [{\hat{d}}_{j}\left(x_{1},x_{2},x_{3},\text{…},x_{N}\right)]}{\partial x_{i}}=\frac{\partial [{\hat{d}}_{j}\left(x_{i}\right)]}{\partial x_{i}}+\frac{\partial [d_{combi}\left(x_{1},x_{2},x_{3},\text{…},x_{N}\right)]}{\partial x_{i}}\\ & \frac{\partial^{2}\left[{\hat{d}}_{j}\left(x_{1},x_{2},x_{3},\text{…},x_{N}\right)\right]}{\partial {\left[x_{i}\right]}^{2}}=\frac{\partial^{2}\left[{\hat{d}}_{j}\left(x_{i}\right)\right]}{\partial {\left[x_{i}\right]}^{2}}+\frac{\partial^{2}\left[d_{combi}\left(x_{1},x_{2},x_{3},\text{…},x_{N}\right)\right]}{\partial {\left[x_{i}\right]}^{2}} \end{array}\right.,\\ & & i=1,2,3,\text{…},N \end{array}$$

#### 2.2.1 Partial Derivatives Calculation of Single Variable Terms

If a function learned by the neural network does not include any composite terms, that is *d*_*combi*_(*x*_1_, *x*_2_, *x*_3_, …, *x*_*N*_) = 0, the following considerations are necessary when calculating partial derivatives:

(17) $$\frac{dX_{i}}{dt}\left(t\right)=\lim_{\varepsilon \to 0^{+}}\;\frac{X_{i}\left(t\right)-X_{i}\left(t-\varepsilon \right)}{\varepsilon },i=1,2,3,\text{…},N$$

where 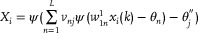, which is the output of the first N neural networks in Equation (14).

**Figure 3:**
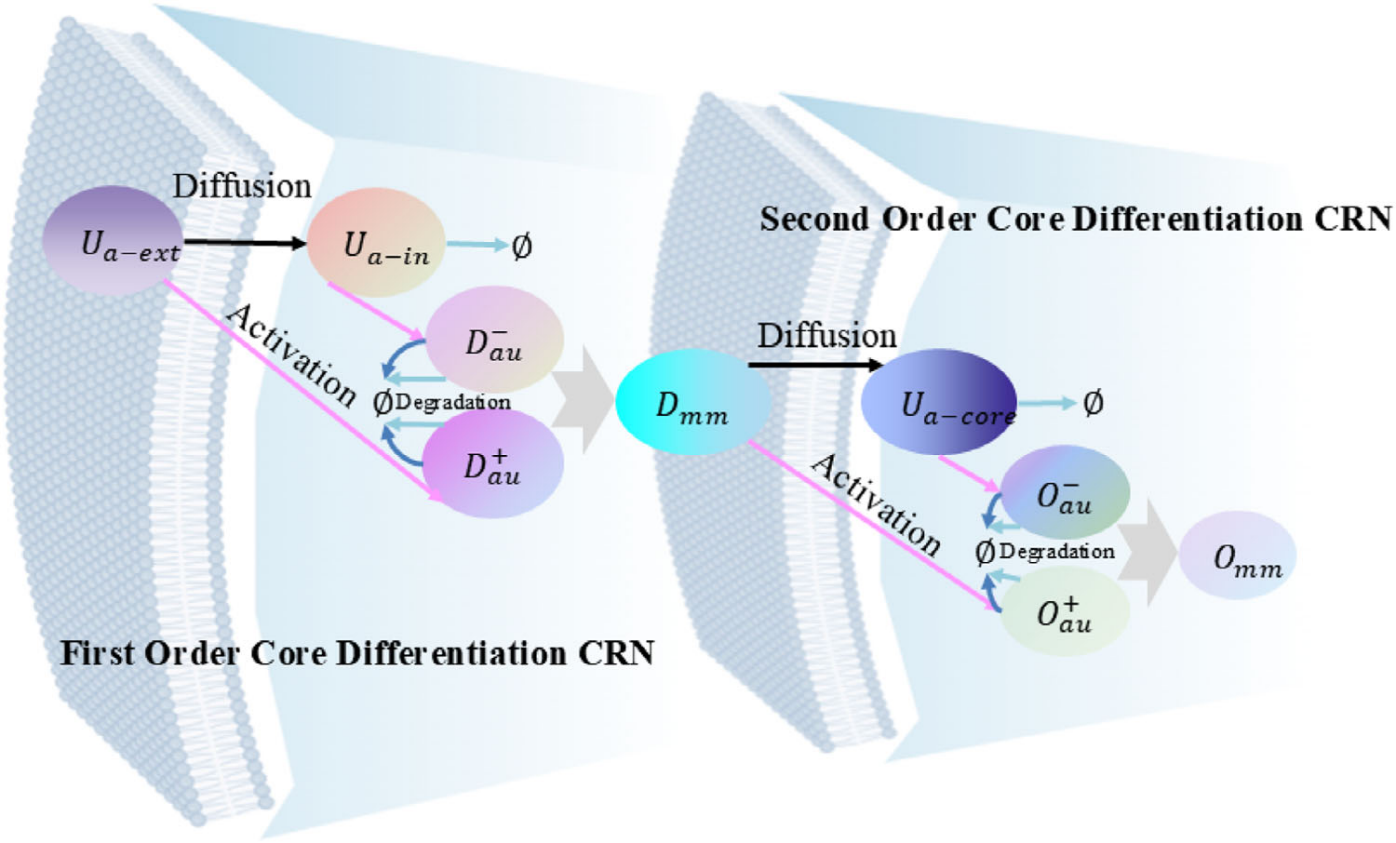
Architecture diagram of the partial differential derivation solution based on membrane diffusion theory.

The partial differential derivative process based on the membrane diffusion method shown in **Figure** **3** consists of two stages: the first‐order partial derivative and the second‐order partial derivative. The second‐order derivative calculation is obtained by performing a secondary derivative calculation based on the result of the first‐order derivative calculation.

Consider the first‐order derivative CRN process based on membrane diffusion, which consists of one input, an intermediate species, and two outputs. The external input *U*_*a*‐*ext*_ exists in large quantities outside the membrane, so its concentration remains unaffected by dynamics. Once it passes through the membrane, it is labeled as *U*_*a*‐*in*_. Substance *U*_*a*‐*ext*_ activates substance $D_{au}^{+}$, and substance *U*_*a*‐*in*_ activates substance $D_{au}^{-}$. In addition, a rapid annihilation reaction removes $D_{au}^{+}$ and $D_{au}^{-}$.

The first‐order core derivative CRN can be composed of exactly the following 12 reactions:

(18) $$\begin{array}{ccc} & & U_{a-ext}\overset{k_{diff}}{\to }U_{a-ext}+U_{a-in},U_{a-in}\overset{k_{diff}}{\to }⌀\\ & & U_{a-ext}\overset{k,k_{diff}}{\to }U_{a-ext}+D_{au}^{+},D_{au}^{+}\overset{k}{\to }⌀\\ & & U_{a-in}\overset{k,k_{diff}}{\to }U_{a-in}+D_{au}^{-},D_{au}^{-}\overset{k}{\to }⌀\\ & & D_{au}^{+}+D_{au}^{-}\overset{k_{fast}}{\to }⌀\\ & & D_{au}^{+}\overset{\alpha_{a}}{\to }D_{au}^{+}+U_{mm},D_{au}^{-}\overset{\beta_{a}}{\to }D_{au}^{-}+U_{nn}\\ & & U_{mm}\overset{\delta_{a}}{\to }⌀,U_{nn}\overset{\delta_{a}}{\to }⌀,U_{mm}+U_{nn}\overset{\gamma_{a}}{\to }⌀ \end{array}$$

By applying mass action kinetics to the initial seven reactions outlined in Equation (18), the following outcomes can be derived:

(19) $$\begin{array}{cc} & \frac{dU_{a-in}}{dt}=k_{diff}\left(U_{a-ext}-U_{a-in}\right)\\ & \frac{dU_{au}^{+}}{dt}=k*k_{diff}*U_{a-ext}-k*U_{au}^{+}-k_{fast}*U_{au}^{+}*U_{au}^{-}\\ & \frac{dU_{au}^{-}}{dt}=k*k_{diff}*U_{a-in}-k*U_{au}^{-}-k_{fast}*U_{au}^{+}*U_{au}^{-} \end{array}$$

Assuming $U_{au}=U_{au}^{+}-U_{au}^{-}$, Equation (20) can be obtained from Equation (19):

(20) $$\begin{array}{cc} & \frac{dU_{au}}{dt}=\frac{dU_{au}^{+}}{dt}-\frac{dU_{au}^{-}}{dt}\\ & \;=k*k_{diff}\left(U_{a-ext}-U_{a-in}\right)-k\left(U_{au}^{+}-U_{au}^{-}\right)\\ & \;=k*\left(\frac{U_{a-ext}-U_{a-in}}{\frac{1}{k_{diff}}}-U_{au}\right) \end{array}$$

As input, a sine wave offset to ensure positive value. CRNs of the sine wave *U*_*a*‐*ext*_(*t*) = 1 + sin (*t*) can be expressed:

(21) $$\begin{array}{ccc} & & A_{p}\overset{l}{\to }A_{p}+B_{p}+U_{a-ext}\\ & & A_{m}\overset{l}{\to }A_{m}+B_{m}+U_{b-ext}\\ & & B_{m}\overset{l}{\to }A_{p}+B_{m}\\ & & B_{p}\overset{l}{\to }A_{m}+B_{p}\\ & & B_{m}+B_{p}\overset{fast}{\to }⌀\\ & & A_{m}+A_{p}\overset{fast}{\to }⌀\\ & & U_{a-ext}+U_{b-ext}\overset{fast}{\to }⌀ \end{array}$$

**Figure 4:**
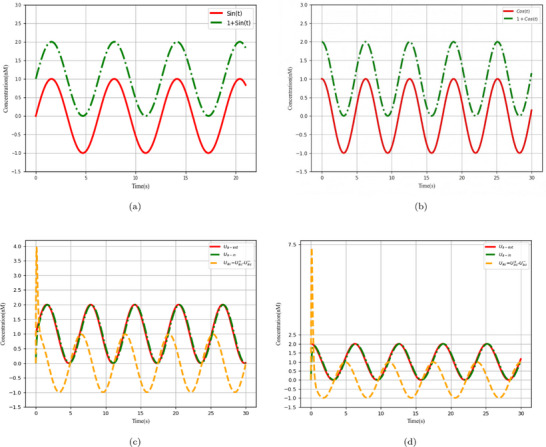
CRN simulation and derivative calculation of equation 1+Sin(t) and 1+Cos(t). a) CRNs representations of the equation 1+Sin(t), b) CRNs representations of the equation 1+Cos(t), c) Simulation results of the derivative of equation 1+Sin(t), and d) Simulation results of the derivative of equation 1+Cos(t).

The parameters *l* and *fast* represent the catalytic reaction rate and annihilation reaction rate, respectively, and are set to *l* = 1.0 and *fast* = 10^5^. **Figure** **4**a depicts the sine offset resulting from Equation (21); the simulated first‐order derivative of the sine wave function using the membrane diffusion theory is shown in Figure 4c.

And a cosine wave offset to remain positive as input. CRNs of the cosine wave 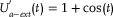 can be expressed as:

(22)

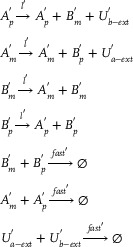

The parameters 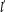 and 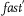 represent the catalytic reaction rate and annihilation reaction rate, respectively, and are set to 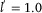 and 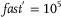. Figure 4b illustrates the cosine outcome represented by Equation (22), while Figure 4d displays the simulation results of the first‐order derivative of the cosine function using the membrane diffusion theory.

Remark 2 Assuming $U_{a-\mathrm{in}}\left(t\right)=\int_{0}^{t}\;k_{\mathrm{diff}}\left(U_{a-\mathrm{ext}}\left(s\right)-U_{a-\mathrm{in}}\left(s\right)\right)\;ds$, the first formula of Equation (19) can be symbolically integrated and transformed into

(23) $$U_{a-in}\left(t\right)=k_{diff}\int_{0}^{t}U_{a-ext}\left(s\right)ds-k_{diff}\int_{0}^{t}U_{a-in}\left(s\right)ds$$

Let *u* = *t* − *s*, then *s* = *t* − *u*, *ds* = −*du*. When *s* = 0, *u* = *t* is attained, and when *s* = *t*, *u* = 0 is produced. Then Equation (23) can be rewritten as:

(24) $$U_{a-in}\left(t\right)=k_{diff}\int_{t}^{0}U_{a-ext}\left(t-u\right)\left(-du\right)-k_{diff}\int_{0}^{t}U_{a-in}\left(s\right)ds$$

Using the property that exp(−*k*_*diff*_ *s*) tends to 0 at *s* → ∞, the upper limit of the integral is extended to ∞.

(25) $$U_{a-in}\left(t\right)=k_{diff}*\int_{0}^{\infty }\exp\left(-k_{diff}s\right)U_{a-ext}\left(t-s\right)ds$$

If *k*_*diff*_ → ∞, then $\tau =\frac{1}{k_{diff}}\to 0$. Assuming *U*_*a*‐*ext*_ is infinitely differentiable, Taylor expansion of *U*_*a*‐*ext*_(*t* −*s*) at *s* = 0 gives the following results:

(26) $$U_{a-ext}\left(t-s\right)=\sum_{n=0}^{\infty }\frac{{\left(-s\right)}^{n}}{n!}U_{a-ext}^{\left(n\right)}\left(t\right)$$

Substituting Equation (26) into Equation (25), equation(27) can be obtained.

(27) $$\begin{array}{cc} U_{a-in}\left(t\right)=\; & \int_{0}^{\infty }k_{diff}*\exp\left(-k_{diff}s\right)\sum_{n=0}^{\infty }\frac{{\left(-s\right)}^{n}}{n!}U_{a-ext}^{\left(n\right)}\left(t\right)ds\\ =\; & \sum_{n=0}^{\infty }\frac{k_{diff}}{n!}U_{a-ext}^{\left(n\right)}\left(t\right)\int_{0}^{\infty }{\left(-s\right)}^{n}*\exp\left(-k_{diff}s\right)ds \end{array}$$

For the integral $I_{n}=\int_{0}^{\infty }{\left(-s\right)}^{n}\exp\left(-k_{diff}s\right)ds$, integration by parts can be utilized to rewrite it. Let *u* = (− *s*)^*n*^, *dv* = exp (− *k*_*diff*_ *s*)*ds*, then $du=-n{\left(-s\right)}^{n-1}ds,v=-\frac{1}{k_{diff}}\exp\left(-k_{diff}s\right)$.

(28) $$\begin{array}{ccc} I_{n} & = & {\left[-{\left(-s\right)}^{n}\frac{1}{k_{diff}}\exp\left(-k_{diff}s\right)\right]}_{0}^{\infty }+\frac{n}{k_{diff}}\int_{0}^{\infty }{\left(-s\right)}^{n-1}\exp\left(-k_{diff}s\right)ds \end{array}$$

When *s* → ∞, ${\left(-s\right)}^{n}\frac{1}{k_{diff}}\exp\left(-k_{diff}s\right)\to 0$; when *S* = 0, ${\left[-{\left(-s\right)}^{n}\frac{1}{k_{diff}}\exp\left(-k_{diff}s\right)\right]}_{0}^{\infty }=0$. According to the above analysis, $I_{n}=\frac{n}{k_{diff}}I_{n-1}$ can be inferred. Equation (29) can be developed further.

(29) $$I_{n}={\left(-1\right)}^{n}{\left(\tau \right)}^{n+1}n,\tau =\frac{1}{k_{diff}}$$

Equation (29) can be substituted into Equation (27), yielding the following result:

(30)

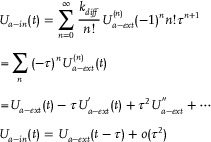

Substituting Equation (30) into Equation (20), Equation (31) can be obtained:

(31) $$\begin{array}{cc} & \frac{dU_{au}}{dt}=\frac{dU_{au}^{+}}{dt}-\frac{dU_{au}^{-}}{dt}\\ & \;=k*k_{diff}\left(U_{a-ext}-U_{a-in}\right)-k\left(U_{au}^{+}-U_{au}^{-}\right)\\ & \;=k*k_{diff}\left(U_{a-ext}-\left(U_{a-ext}\left(t-\tau \right)+o\left(\tau^{2}\right)\right)\right)-kU_{au} \end{array}$$

Further simplification yields equation(32).

(32) $$\frac{dU_{au}}{dt}=k*k_{diff}(U_{a-ext}-\left(U_{a-ext}\left(t-\tau \right)\right)-kU_{au}+o\left(\tau \right)$$

Introducing a delay ε and setting $\varepsilon =\frac{1}{k}$, the following result can be achieved:

(33) $$D\left(t\right)=\frac{U_{a-ext}\left(t-\varepsilon \right)-U_{a-ext}\left(t-\tau -\varepsilon \right)}{\tau }+o\left(\tau \right)+o\left(\varepsilon^{2}\right)$$

*o*(τ) is the high‐order infinitesimal term related to τ generated in the previous simplification process, and *o*(ε^2^) is the high‐order infinitesimal term generated by the approximation related to ε.

**Figure 5:**
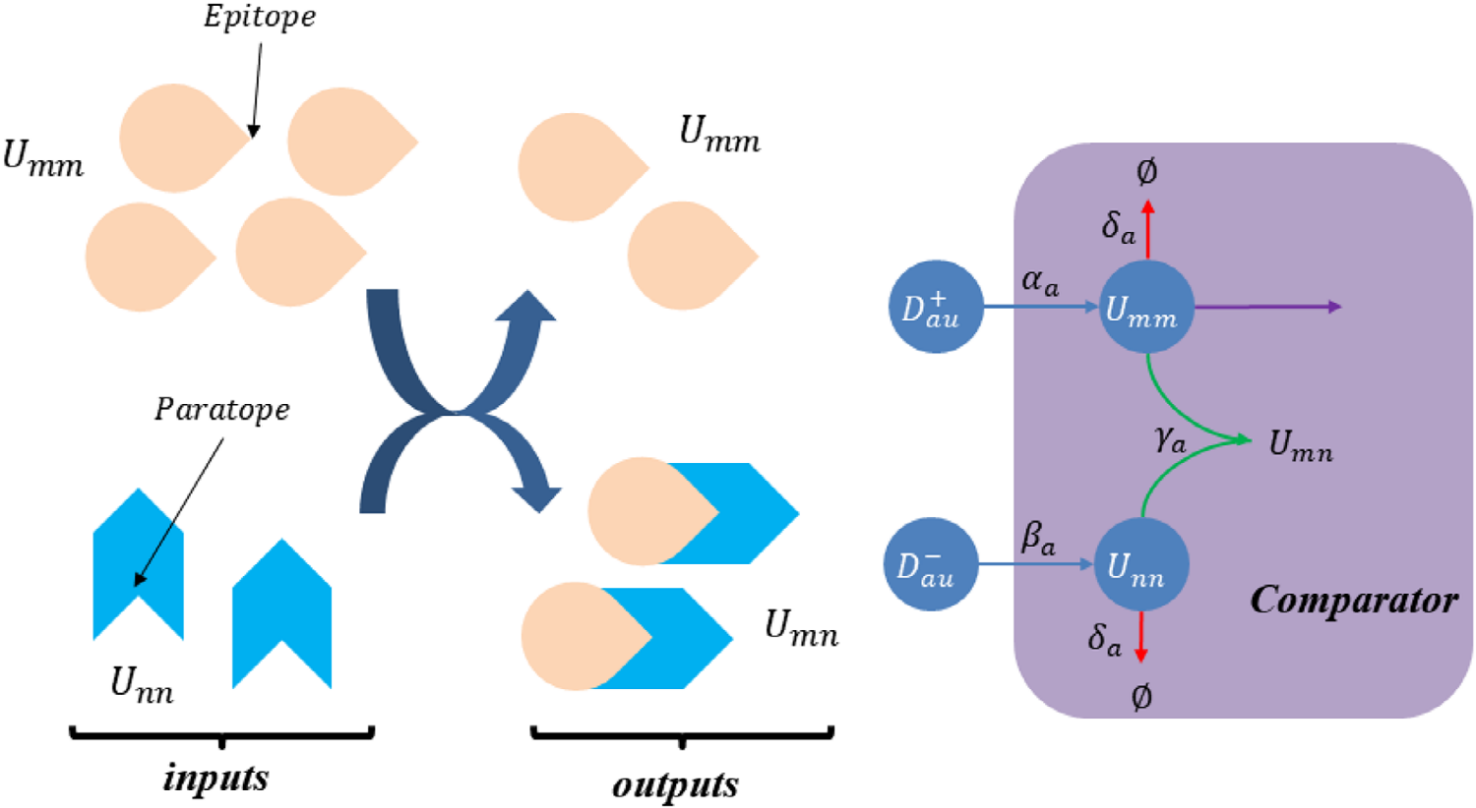
Block diagram of the subtraction operator.

As illustrated in **Figure** **5**, the subtraction operation consists of the last five reactions in Equation (18), and the corresponding ordinary differential equations (ODEs) can be mathematically derived by

(34) $$\begin{array}{cc} & \frac{dU_{mm}}{dt}=\alpha_{a}*D_{au}^{+}-\delta_{a}*U_{mm}-\gamma_{a}*U_{mm}*U_{nn}\\ & \frac{dU_{nn}}{dt}=\beta_{a}*D_{au}^{-}-\delta_{a}*U_{nn}-\gamma_{a}*U_{mm}*U_{nn} \end{array}$$

Assuming that the reaction rate α_*a*_ = β_*a*_ = δ_*a*_ and *U*_*nn*_ → 0, when the reaction system described by Equation (18) achieves steady‐state equilibrium, the left‐hand side terms $\frac{dU_{mm}}{dt}$ and $\frac{dU_{nn}}{dt}$ of Equation (34) can be set to zero, resulting in the following:

(35) $$U_{mm}=D_{au}^{+}-D_{au}^{-}$$

Combining Equation (20), Equation (36) can be obtained:

(36) $$\begin{array}{cc} & \frac{dU_{mm}}{dt}=\frac{dU_{au}^{+}}{dt}-\frac{dU_{au}^{-}}{dt}\\ & \;=k*k_{diff}\left(U_{a-ext}-U_{a-in}\right)-k\left(U_{au}^{+}-U_{au}^{-}\right)\\ & \;=k*\left(\frac{U_{a-ext}-U_{a-in}}{\frac{1}{k_{diff}}}-U_{mm}\right) \end{array}$$

According to Equation (36), when the reaction system (18) achieves a steady state, the first‐order partial differential result *U*_*mm*_ of substance *U*_*a*‐*ext*_ can be produced:

(37) $$U_{mm}=\frac{U_{a-ext}-U_{a-in}}{\frac{1}{k_{diff}}}$$

Combining Equations (20), (30) and (37), the following result can be obtained:

(38) $$\begin{array}{cc} & \frac{dU_{a-ext}}{dt}\left(t\right)=\lim_{\varepsilon \to 0^{+}}\;\frac{U_{a-ext}\left(t\right)-U_{a-ext}\left(t-\varepsilon \right)}{\varepsilon }\\ & \;\approx \frac{U_{a-ext}-U_{a-in}}{\frac{1}{k_{diff}}}=U_{mm} \end{array}$$

where $\varepsilon =\frac{1}{k_{diff}}$. Similarly, the second‐order derivative output can be derived as:

(39) $$\begin{array}{cc} & \frac{dO_{mm}}{dt}=\frac{dO_{au}^{+}}{dt}-\frac{dO_{au}^{-}}{dt}\\ & \;=k*k_{diff}\left(D_{mm}-U_{a-core}\right)-k\left(O_{au}^{+}-O_{au}^{-}\right)\\ & \;=k*\left(\frac{D_{mm}-U_{a-core}}{\frac{1}{k_{diff_{2}}}}-O_{mm}\right) \end{array}$$

The parameter *k*_*diff*2_ denotes the rate in the second‐order derivative stage. Equation (38) yields the first‐order partial differential result *O*_*mm*_ of substance (parameter) *U*_*a*‐*in*_, as well as the second‐order partial differential result of *U*_*a*‐*ext*_:

(40) $$O_{mm}=\frac{D_{mm}-U_{a-core}}{\frac{1}{k_{diff_{2}}}}$$

The differential CRN aims to compute a function *f* from an unknown input signal *U*_*a*‐*ext*_. Compute *f*(*U*_*a*‐*ext*_(*t*)) for a given function *f*. However, the differential CRN only provides for an approximation of the input signal's derivative. The first‐order calculation equation can be expressed as:

(41) $$\begin{array}{ccc} & & \left\{\begin{array}{cc} & \frac{dY}{dt}=f^{\prime }\left(U_{a-ext}\left(t\right)\right)\frac{dU_{a-ext}}{dt}\\ & \;=f^{\prime }\left(U_{a-ext}\left(t\right)\right)*\lim_{\varepsilon \to 0^{+}}\;\frac{U_{a-ext}\left(t\right)-U_{a-ext}\left(t-\varepsilon \right)}{\varepsilon }\\ & \;\approx f^{\prime }\left(U_{a-ext}\left(t\right)\right)*\frac{U_{a-ext}-U_{a-in}}{\frac{1}{k_{diff}}}\\ & \;=f^{\prime }\left(U_{a-ext}\left(t\right)\right)*U_{mm} \end{array}\right.,\\ & & \;Y\left(0\right)=f\left(U_{a-ext}\left(0\right)\right)\varepsilon =\frac{1}{k_{diff}} \end{array}$$

The second‐order calculation equation can be described by Equation (42).

(42) $$\begin{array}{ccc} & & \left\{\begin{array}{cc} & \frac{d^{2}Y}{dt^{2}}=\frac{d}{dt}\left(f^{\prime }\left(U_{a-ext}\left(t\right)\right)\right)\cdot \frac{dU_{a-ext}}{dt}+f^{\prime }(U_{a-ext}\left(t\right)\cdot \frac{d}{dt}\left(\frac{dU_{a-ext}}{dt}\right)\\ & \;=\left(f^{\prime \prime }\left(U_{a-ext}\left(t\right)\right)\cdot \frac{dU_{a-ext}}{dt}\right)\cdot \frac{dU_{a-ext}}{dt}+f^{\prime }\left(U_{a-ext}\left(t\right)\right)\cdot \frac{d}{dt}\left(\frac{dU_{a-ext}}{dt}\right)\\ & \;=f^{\prime \prime }\left(U_{a-ext}\left(t\right)\right)\cdot {\left(\frac{dU_{a-ext}}{dt}\right)}^{2}+f^{\prime }(U_{a-ext}\left(t\right)\cdot \frac{d^{2}U_{a-ext}}{dt^{2}} \end{array}\right.,\\ & & Y\left(0\right)=f(U_{a-ext}\left(0\right)) \end{array}$$

**Figure 6:**
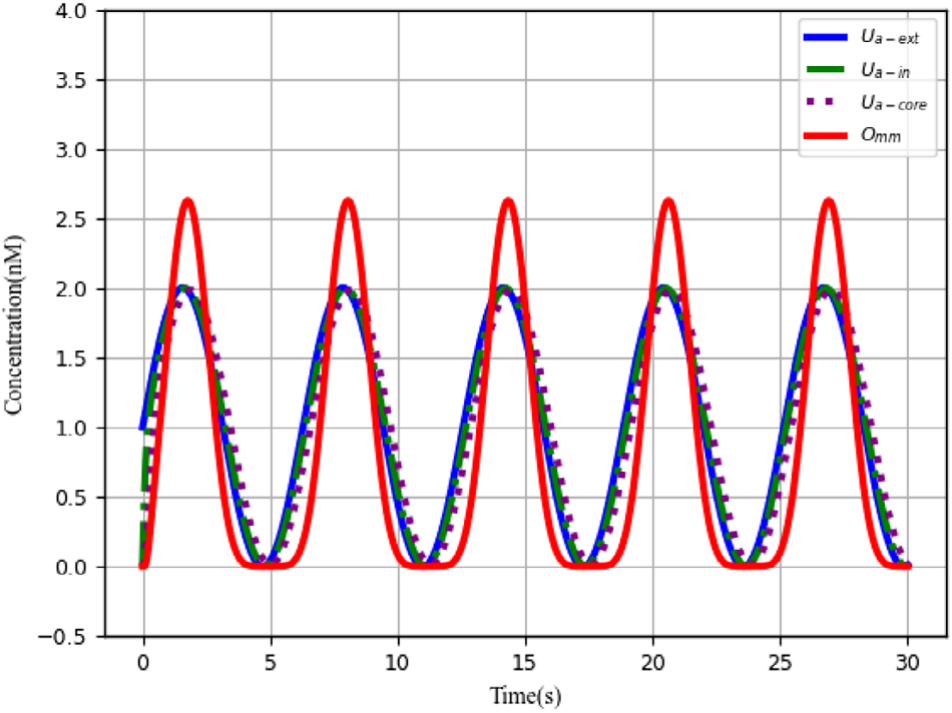
Calculation of higher‐order derivatives based on membrane diffusion theory.

Let us consider the cube function depicted in **Figure** 6:

(43) $$\left\{\begin{array}{cc} & \frac{dY}{dt}=U_{a-ext}^{2}*U_{mm}\\ & \frac{d^{2}Y}{dt^{2}}=2U_{a-ext}*U_{mm}^{2}+U_{a-ext}^{2}*O_{mm} \end{array}\right.$$

And a sine wave offset to remain positive as input: *U*_*a*‐*ext*_(*t*) = 1 + sin (*t*). Theoretical verification of the high‐order derivative results of function 1 + sin (*t*) based on membrane diffusion theory:

(44) $$\begin{array}{cc} & x(t)=1+\sin(t)\\ & x^{\prime }\left(t-\tau \right)=\cos\left(t-\tau \right)=cos(t\text{)+}\tau *\sin\left(t\right)+O\left({\tau_{1}}^{2}\right)\\ & x^{\prime \prime }\left(t-\tau \right)=-\sin\left(t-\tau \right)=-\text{sin(}t\text{) +}\tau *\cos\left(t\right)+O\left({\tau_{2}}^{2}\right) \end{array}$$

Then compute the output to the second order and first order:

(45) $$\begin{array}{ccc} y^{\prime }\left(t\right) & = & \int 2x\left(s\right)*{x^{\prime }}^{2}\left(s-\tau \right)ds+x^{2}\left(s\right)*x^{\prime \prime }\left(s-\tau \right)ds\\ & = & \int 2\left(1+\sin\left(s\right)\right)*{\left[\cos(s)+\tau *\sin(s)\right]}^{2}ds\\ & & +\int {\left(1+\sin(s)\right)}^{2}*\left[-\sin\left(s\right)+\tau *\cos\left(s\right)\right]ds\\ & = & \int 2\left(1+\sin\left(s\right)\right)*\cos^{2}\left(s\right)ds+\int {\left(1+\sin(s)\right)}^{2}*\left[-\sin\left(s\right)\right]ds\;\\ & & +\int 2\left(1+\sin\left(s\right)\right)*\left[\cos\left(s\right)*2\tau *\sin\left(s\right)+\tau^{2}*\sin^{2}\left(s\right)\right]ds\\ & & +\int {\left(1+\sin(s)\right)}^{2}*\tau *\cos\left(s\right)]ds\\ & = & {\left[1+\sin(t)\right]}^{2}\text{*cos(}t\text{)+}\int 2\left(1+\sin\left(s\right)\right)*[\cos\left(s\right)*2\tau *\sin\left(s\right)\\ & & +\tau^{2}*\sin^{2}\left(s\right)]ds+\int {\left(1+\sin(s)\right)}^{2}*\tau *\cos\left(s\right)]ds\\ y^{\prime }\left(t\right) & \approx & {\left[1+\sin(t)\right]}^{2}\text{*cos(}t\text{)+}\tau t\\ y(t) & = & \int {\left[1+\sin(t)\right]}^{2}\text{*cos(}t\text{)d}s+\;\int \tau tds\\ y(t) & \approx & \frac{1}{3}*{\left[1+\sin(t)\right]}^{3} \end{array}$$

#### 2.2.2 Partial Derivatives Calculation of Compound Terms

If the function learned by the neural network does not contain any composite terms, i.e., *d*_*combi*_(*x*_1_, *x*_2_, *x*_3_, …, *x*_*N*_) ≠ 0, the mutual influence between distinct variables in the composite terms needs to be addressed while calculating partial derivatives.

Case 1 If *d*_*combi*_(*x*, *t*) = *xt* + *x* ^2^ *t* ^2^, the corresponding CRNs can be described by:

(46) $$\begin{array}{cc} & \left\{\begin{array}{cc} & X+T\overset{k}{\to }X+T+B\\ & B\overset{k}{\to }⌀ \end{array}\right.\\ & \left\{\begin{array}{cc} & 2X+2T\overset{k}{\to }2X+2T+C\\ & C\overset{k}{\to }⌀ \end{array}\right.\\ & \left\{\begin{array}{cc} & B\overset{k}{\to }B+D\\ & C\overset{k}{\to }C+D\\ & D\overset{k}{\to }⌀ \end{array}\right. \end{array}$$

Using mass action kinetics, the ordinary differential equations (ODEs) of Equation (46) could be derived:

(47) $$\begin{array}{cc} & \frac{dB}{dt}=k*X*T-k*B\\ & \frac{dC}{dt}=k*X^{2}*T^{2}-k*C\\ & \frac{dD}{dt}=k*B+k*C-k*D \end{array}$$

When the reaction system (46) reaches a steady state, the terms $\frac{dB}{dt}$, $\frac{dC}{dt}$, and $\frac{dD}{dt}$ on the left side of Equation (47) can be zeroed, resulting in Equation (48).

(48) $$\begin{array}{cc} & B=XT\\ & C=X^{2}T^{2}\\ & D=B+C=XT+X^{2}T^{2} \end{array}$$

The partial differential derivation principle can yield the following results:

(49) $$\frac{\partial_{combi}\left(x,t\right)}{\partial x}=\frac{1}{x}*xt+\frac{2}{x}*x^{2}t^{2}$$

The first‐order partial derivative of a single variable *x* computed via CRNs is given as:

(50) $$\begin{array}{cc} & div\left[B,X,E\right]:\left\{\begin{array}{cc} & B\overset{k}{\to }B+E\\ & X+E\overset{k}{\to }X \end{array}\right.\\ & div\left[C,X,F\right]:\left\{\begin{array}{cc} & C\overset{2k}{\to }C+F\\ & X+F\overset{k}{\to }X \end{array}\right.\\ & add\left[E,F,G\right]\left\{\begin{array}{cc} & E\overset{k}{\to }E+G\\ & F\overset{k}{\to }F+G\\ & G\overset{k}{\to }⌀ \end{array}\right. \end{array}$$

The ODEs of Equation (50) are indicated as:

(51) $$\begin{array}{cc} & \frac{dE}{dt}=k*B-k*X*E\\ & \frac{dF}{dt}=2k*C-k*X*F\\ & \frac{dG}{dt}=k*E+k*F-k*G \end{array}$$

When the reaction system (50) reaches a steady state, it can be obtained:

(52) $$\begin{array}{cc} & E=\frac{B}{X}\\ & F=\frac{2C}{X}\\ & G=E+F=\frac{B}{X}+\frac{2C}{X}=\frac{1}{X}*X*T+\frac{2}{X}*X^{2}*T^{2} \end{array}$$

Obviously, the outcome of Equation (52) is consistent with that of Equation (49). Furthermore, the CRNs generated using the first‐order partial derivatives of variables *x* and *t* could be represented as:

(53) $$\begin{array}{ccc} & & div\left[B,\left(X*T\right),K\right]:\left\{\begin{array}{cc} & B\overset{k}{\to }B+K\\ & X+T+K\overset{k}{\to }X+T \end{array}\right.\\ & & div\left[C,\left(X*T\right),L\right]:\left\{\begin{array}{cc} & C\overset{2*2*k}{\to }C+L\\ & X+T+L\overset{k}{\to }X+T \end{array}\right.\\ & & add\left[H,L,M\right]\left\{\begin{array}{cc} & K\overset{k}{\to }K+M\\ & L\overset{k}{\to }L+M\\ & M\overset{k}{\to }⌀ \end{array}\right. \end{array}$$

Remark 3 Assuming that *d*_*combi*_(*x*, *t*) = a*xt + b*xt ^2^ + c*x ^2^ *t* ^2^ + d*x ^3^ *t* + e*x ^3^ *t* ^2^ + f*x ^3^ *t* ^3^ is the composite term, the following result could be reached using the partial differential derivation module.

(54) $$\begin{array}{ccc} \frac{\partial y_{combi}\left(x,t\right)}{\partial x} & = & x\left(a*t+b*t^{2}\right)+c*x^{2}t^{2}+x^{3}\left(d*t+e*t^{2}+f*t^{3}\right)\\ & = & \frac{1}{x}x\left(a*t+b*t^{2}\right)+\frac{2}{x}c*x^{2}t^{2}+\frac{3}{x}x^{3}\left(d*t+e*t^{2}+f*t^{3}\right)\\ \frac{\partial y_{combi}\left(x,t\right)}{\partial t} & = & t(a*x+d*x^{3}\text{)+}t^{2}\left(b*x+c*x^{2}+e*x^{3}t^{2}\right)+t^{3}\left(f*x^{3}\right)\\ & = & \frac{1}{t}t(a*x+d*x^{3}\text{)+}\frac{2}{t}t^{2}\left(b*x+c*x^{2}+e*x^{3}t^{2}\right)+\frac{3}{t}t^{3}\left(f*x^{3}\right)\\ \frac{\partial y_{combi}\left(x,t\right)}{\partial x\partial t} & = & \frac{1*1}{xt}a*xt+\frac{1*2}{xt}b*xt^{2}+\frac{2*2}{xt}c*x^{2}t^{2}\\ & & +\frac{3*1}{xt}d*x^{3}t+\frac{3*2}{xt}e*x^{3}t^{2}+\frac{3*3}{xt}f*x^{3}t^{3} \end{array}$$

According to the two equations $\frac{\partial y_{combi}\left(x,t\right)}{\partial x}$ and $\frac{\partial y_{combi}\left(x,t\right)}{\partial t}$ in Equation (54), the calculation rule of the first‐order partial derivative with respect to variable *x* or *t* can be derived:

(55) $$\begin{array}{cc} & \frac{E\text{xponent of the variable}\;x\;\text{or}\;t\;\text{in}\;y_{combi-current}}{Variable\;x\;\text{or}\;t}*y_{combi-current} \end{array}$$

The CRNs calculated by the first‐order partial derivative of a single variable *x* can be expressed as:

(56) $$\left\{\begin{array}{cc} & y_{combi-current}\overset{k_{1}}{\to }y_{combi-current}+O\\ & X+O\overset{k_{2}}{\to }X \end{array}\right.$$

where $\frac{k_{1}}{k_{2}}$ is the exponent of the variable *x* in *y*_*combi*‐*current*_.

The CRNs calculated by the first‐order partial derivative of a single variable *t* can be described as:

(57) $$\left\{\begin{array}{cc} & y_{combi-current}\overset{k_{3}}{\to }y_{combi-current}+P\\ & T+P\overset{k_{4}}{\to }T \end{array}\right.$$

where $\frac{k_{3}}{k_{4}}$ is the exponent of the variable *t* in *y*_*combi*‐*current*_. According to equation $\frac{\partial y_{combi}\left(x,t\right)}{\partial x\partial t}$ in Equation (54), calculation rules of the first‐order partial derivatives with respect to variables *x* and *t* can be obtained:

(58) Multiplication of the exponentsof the variablexandtinycombi−currentVariable(x∗t)∗ycombi−current

The CRNs generated using the first‐order partial derivatives of variables *x* and *t* could be represented as:

(59) $$\left\{\begin{array}{cc} & y_{combi-current}\overset{k_{5}}{\to }y_{combi-current}+Q\\ & X+T+Q\overset{k_{6}}{\to }X+T \end{array}\right.$$

where $\frac{k_{5}}{k_{6}}$ represents multiplication of the exponents of the variable *x* and *t* in *y*_*combi*‐*current*_.

### 2.3 CRNs‐Based Objective Function Verification Module

**Figure 7:**
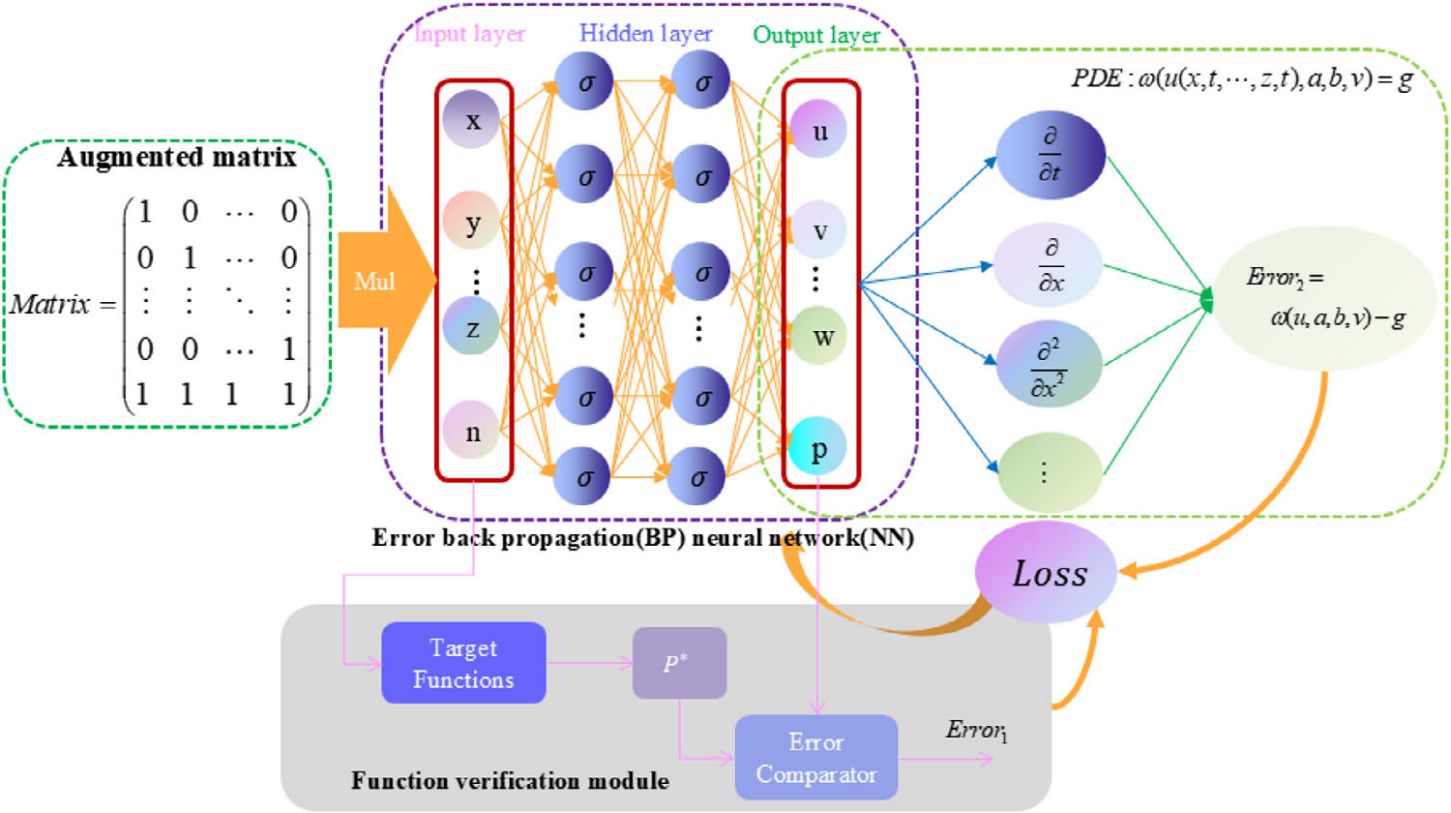
Augmented matrix neural network based on function verification module for solving partial differential equations.

**Figure** **7** depicts the architecture of the augmented matrix neural network for solving partial differential equations, which is based on the objective function verification module. It should be noted that adding a function verification module has two advantages: one is to characterize the learning process of the neural network function and dynamically calculate the real‐time result of the objective function, that is, the signal P* in Figure 7; and the other is to calculate the error value between the neural network output result and the expected result of the objective function, that is, the signal *Error*_1_ in Figure 7.

Assume that the objective function required for the neural network to learn is a typical quadratic function, which is:

(60) $$f\left(x,t\right)=ax^{2}+bt^{2}+cxt$$

The CRNs‐based target function module in the function verification model could be stated as follows:
(61)

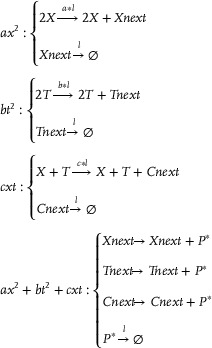

The CRNs‐based error comparator module can be described as:

(62)

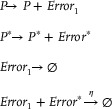

Figure 7 illustrates how the error *Error*_2_ (the equation error) could be derived while computing the partial differential equation. When combined with the actual target error *Error*_1_ as specified by Equation (62), the computation error *Loss* of the entire system could be obtained:

(63)

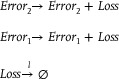




### 2.4 Augmented Matrix Neural Network for Solving Biological Brusselator Partial Differential Equation

#### 2.4.1 Biological Brusselator Partial Differential Equation Based on Diffusion Term

**Figure 8:**
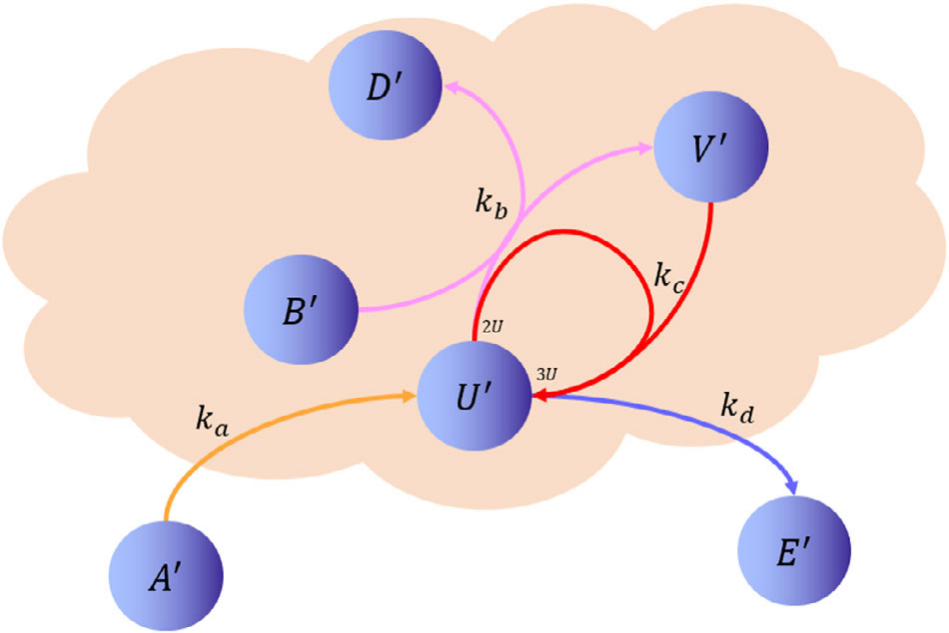
Biological Brusselator reaction model.

The Brusselator model is a classic theoretical model for studying the dissipative structure of nonlinear chemical systems and the formation of biological patterns. The Brusselator model (**Figure** 8) based on CRNs can be described as:
(64)

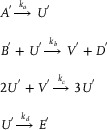




Assuming constant concentrations of substances *A* and *B*, the differential equation representing the concentration changes of intermediate species 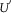 and 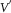 with time and space, including diffusion terms in a one‐dimensional domain, could be described as:
(65)

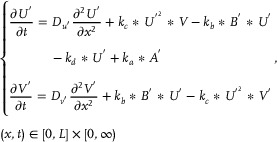

where 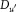 and 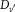 are the diffusion coefficients of 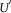 and 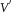 respectively, and *k*_*a*_, *k*_*b*_, *k*_*c*_, *k*_*d*_ is a non‐negative non‐zero reaction rate. Constraints are implemented by applying homogeneous Neumann boundary conditions:
(66)

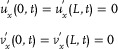

Nonnegative initial conditions:

(67)

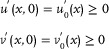




The Brusselator reaction‐diffusion system has no known analytical solution and must be solved numerically. In the absence of diffusion, if Equation (65) obtains a steady‐state solution, the parameters (a, b) must satisfy:

(68)

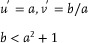




**Figure 9:**
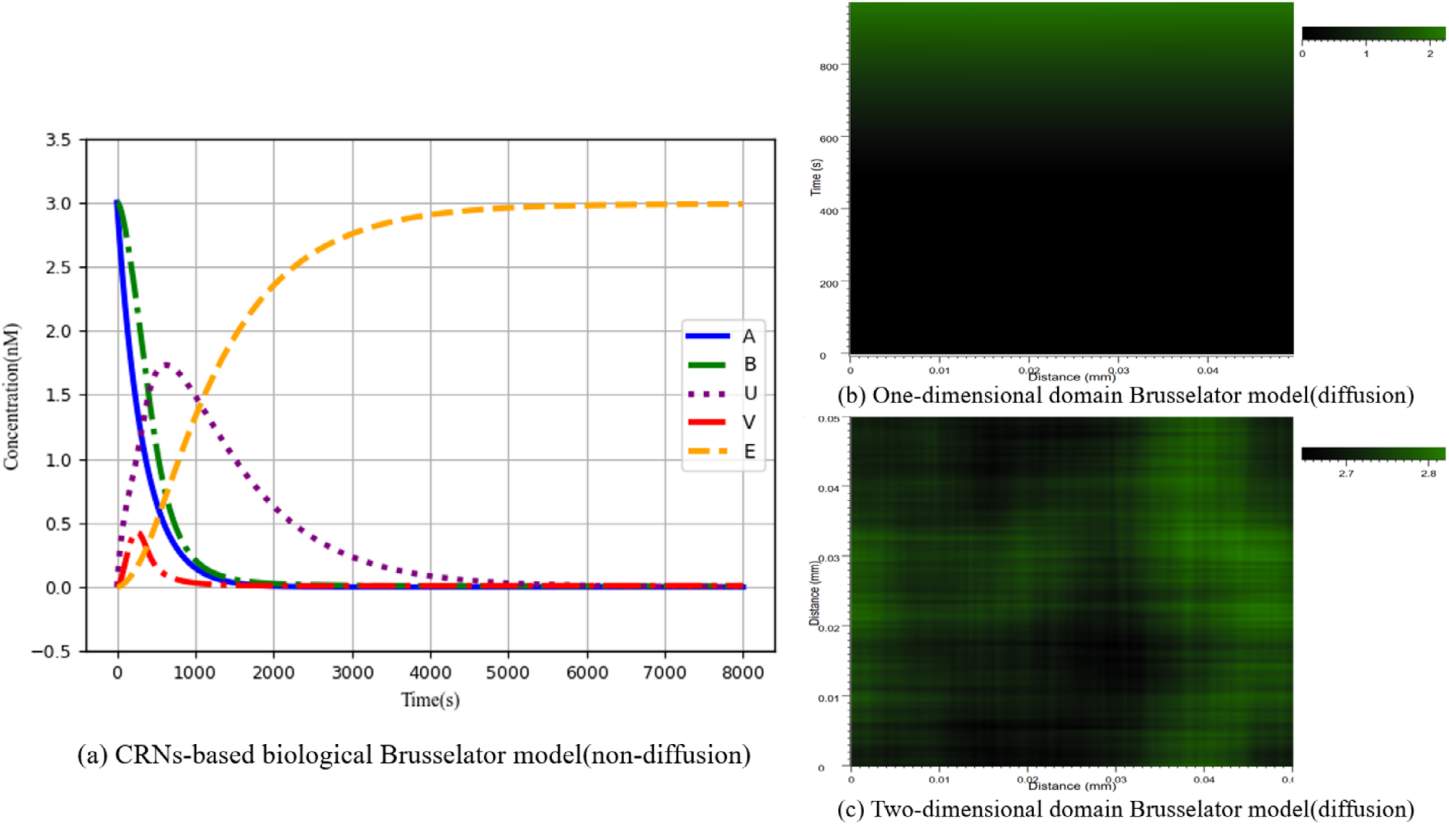
Simulation results of the biological Brusselator model.

The parameters of the biological Brusselator model represented by Equation (64) are established; the starting concentrations of the two input chemicals 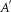 and 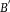 are set to 3*nM*; and the reaction rates are set to *k*_*a*_ = 0.003, *k*_*b*_ = 0.002, *k*_*c*_ = 0.01, and *k*_*d*_ = 0.001. The simulation results are depicted in **Figure** **9**.

#### 2.4.2 Solving the Biological Brusselator Partial Differential Equations

The Brusselator RD (reaction‐diffusion) system described by Equation (65) primarily involves differential calculations, second‐order partial derivative calculations, addition and subtraction operations, and so on. To simplify the partial differential equation solution process, the first equation in Equation (65) is chosen as the research target in the process. The major goal of this research is to solve partial differential equations using the proposed augmented matrix‐based back propagation neural network and the partial differential calculation theory.

(69)

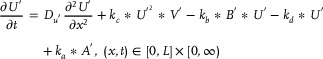




Assume that parameters 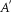, 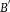, and 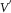 are constants, and parameter 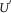 is a function of *x*, *t*, i.e., 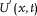, with 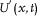 also serving as the output of the augmented matrix‐based back propagation neural network. Equation (69) could be rewritten as:

(70)

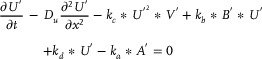

Using 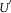, 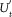 and 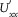 to represent 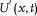, 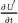 and 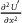 respectively, the CRNs of Equation (70) can be expressed as:

(71)

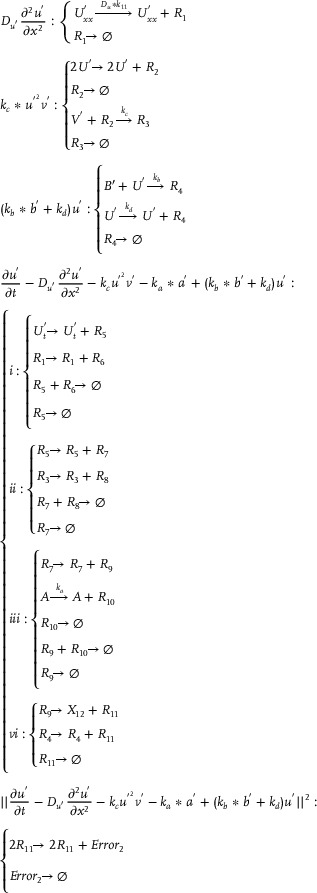

It is worth noting that the actual output species of the chemical system given by Equation (71) is *Error*_2_, whereas Equation (70) produces *R*_11_. The two output species demonstrate the following relationship:

(72) $$\frac{dError_{2}}{dt}=k*R_{11}^{2}-k*Error_{2}$$

When the reaction system is stable, the following result can be obtained through Equation (72).

(73) $$Error_{2}=R_{11}^{2}$$

From the perspective of solving partial differential equations, the final concentration of substance *Error*_2_ can be utilized to represent the equation loss error.

## 3 Implementations With DSD Reaction Networks

In the second section, an augmented matrix neural network (AMN) is built using chemical reaction networks (CRNs) to produce multivariable combination items. The third section implements the membrane diffusion principle and the division calculation principle to calculate the partial derivatives of single‐variable and combination items based on CRNs. In the fourth section, the biological Brusselator model with diffusion terms is studied, the objective function verification module is designed, and the biological Brusselator partial differential equation is solved using an augmented matrix neural network based on the solving‐verified module.

To simplify the description, this section focuses on describing the DNA strand displacement reaction implementation pathways of typical catalytic, degradation, and annihilation reactions in different functional modules.

### 3.1 DNA Implementation of Back Propagation Neural Network Based on Augmented Matrix

**Figure 10:**
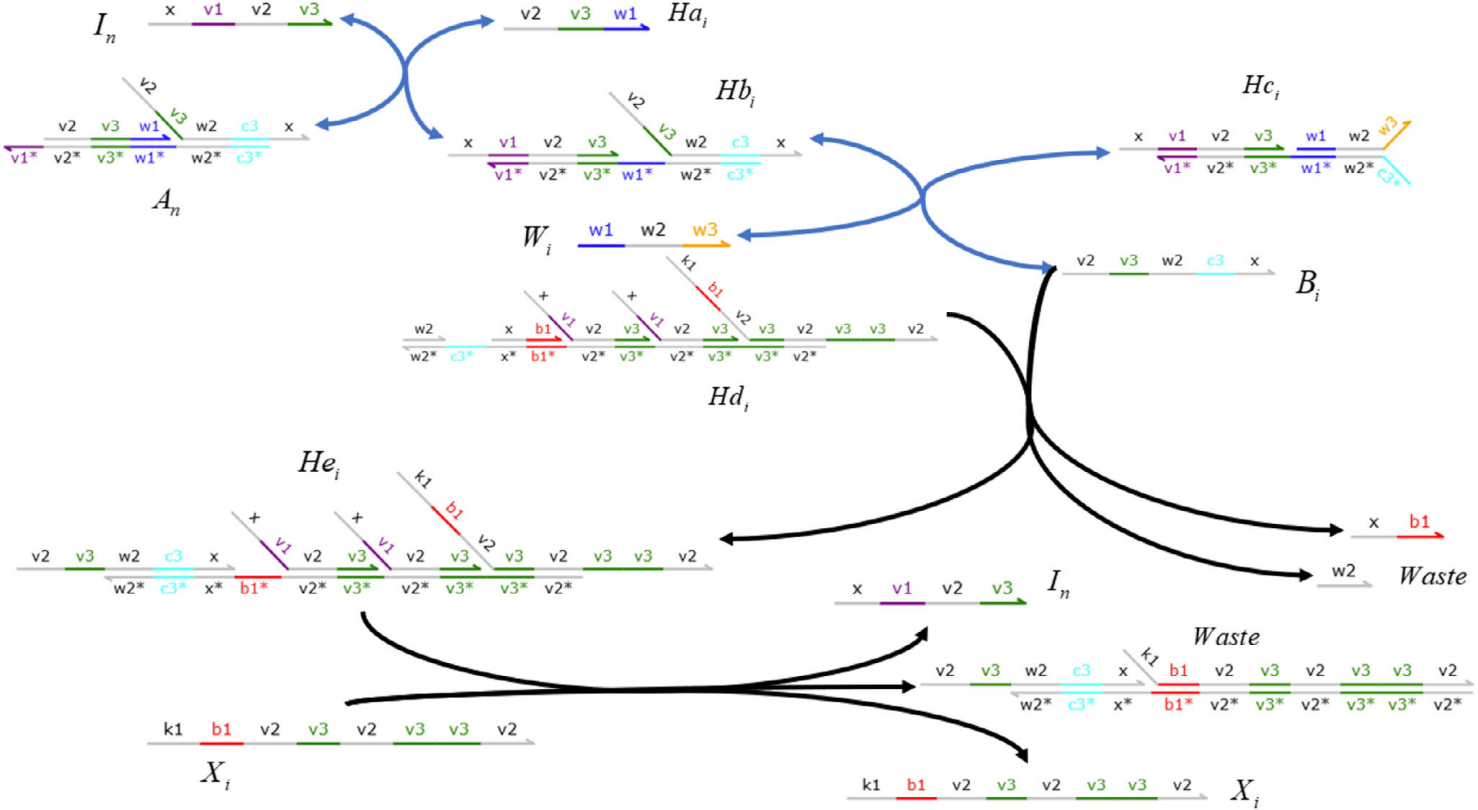
Schematic representation of DNA reactions of Equation (74).

**Catalysis reaction model 1 (Figure** **10** **)**

(74) $$\begin{array}{ccc} & & X_{i}+W_{i}+I_{n}\overset{k_{i}}{\to }X_{i}+2I_{n}\Leftrightarrow \\ & & \;\left\{\begin{array}{cc} & I_{n}+A_{n}\underset{q_{max}}{\overset{q_{i}}{⟷}}Ha_{i}+Hb_{i}\\ & Hb_{i}+W_{i}\underset{q_{max}}{\overset{q_{m}}{⟷}}B_{i}+Hc_{i}\\ & B_{i}+Hd_{i}\overset{q_{max}}{\to }He_{i}+Waste\\ & He_{i}+X_{i}\overset{q_{max}}{\to }X_{i}+2I_{n}+Waste \end{array}\right.,\;q_{i}=k_{i} \end{array}$$

**Figure 11:**
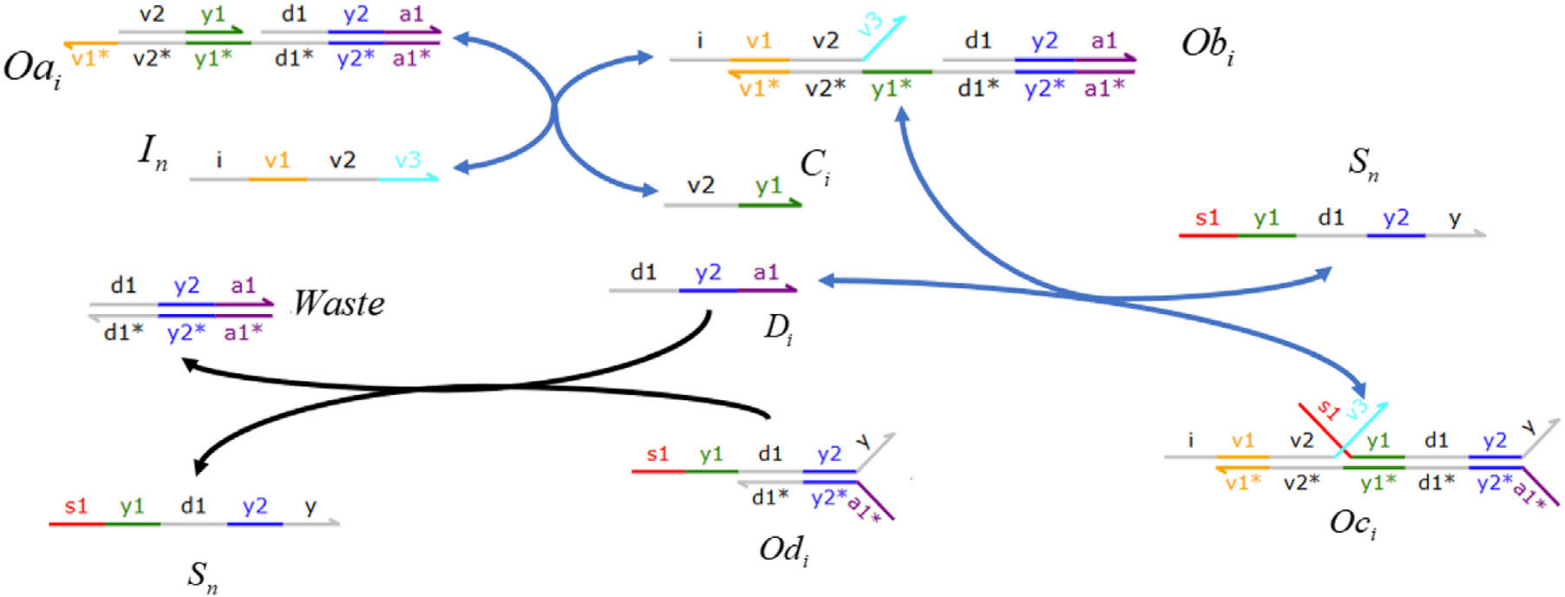
Schematic representation of DNA reactions of Equation (75).

**Degradation reaction model (Figure** **11** **)**

(75) $$I_{n}+S_{n}\overset{k_{i}}{\to }S_{n}\Leftrightarrow \left\{\begin{array}{cc} & I_{n}+Oa_{i}\underset{q_{max}}{\overset{q_{i}}{⟷}}C_{i}+Ob_{i}\\ & Ob_{i}+S_{n}\underset{q_{max}}{\overset{q_{m}}{⟷}}D_{i}+Oc_{i}\\ & D_{i}+Od_{i}\overset{q_{max}}{\to }S_{n}+Waste \end{array}\right.,q_{i}=k_{i}$$

**Figure 12:**
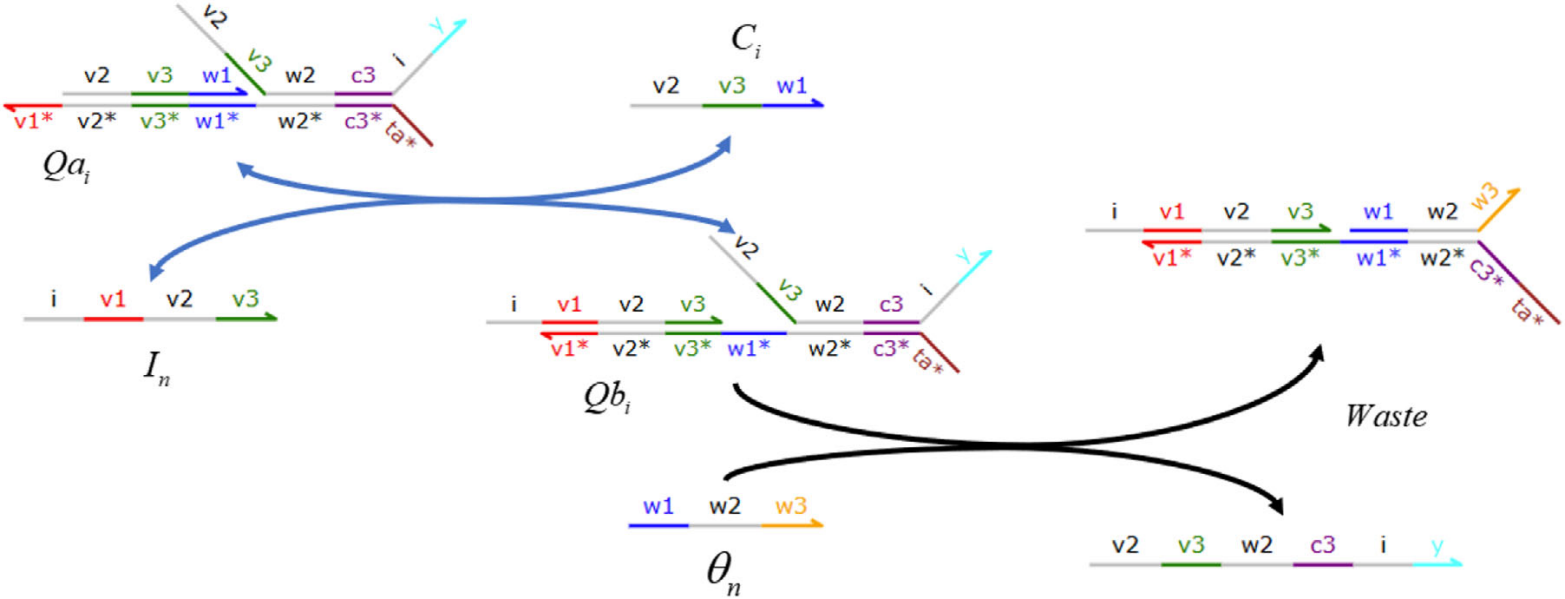
Schematic representation of DNA reactions of Equation (76).

**Annihilation reaction module (Figure** **12** **)**

(76) $$I_{n}+\theta_{n}\overset{k_{i}}{\to }⌀\Leftrightarrow \left\{\begin{array}{cc} & I_{n}+Qa_{i}\underset{q_{max}}{\overset{q_{i}}{⟷}}C_{i}+Qb_{i}\\ & Qb_{i}+\theta_{n}\overset{q_{max}}{\to }Waste \end{array}\right.,q_{i}=k_{i}$$

**Figure 13:**
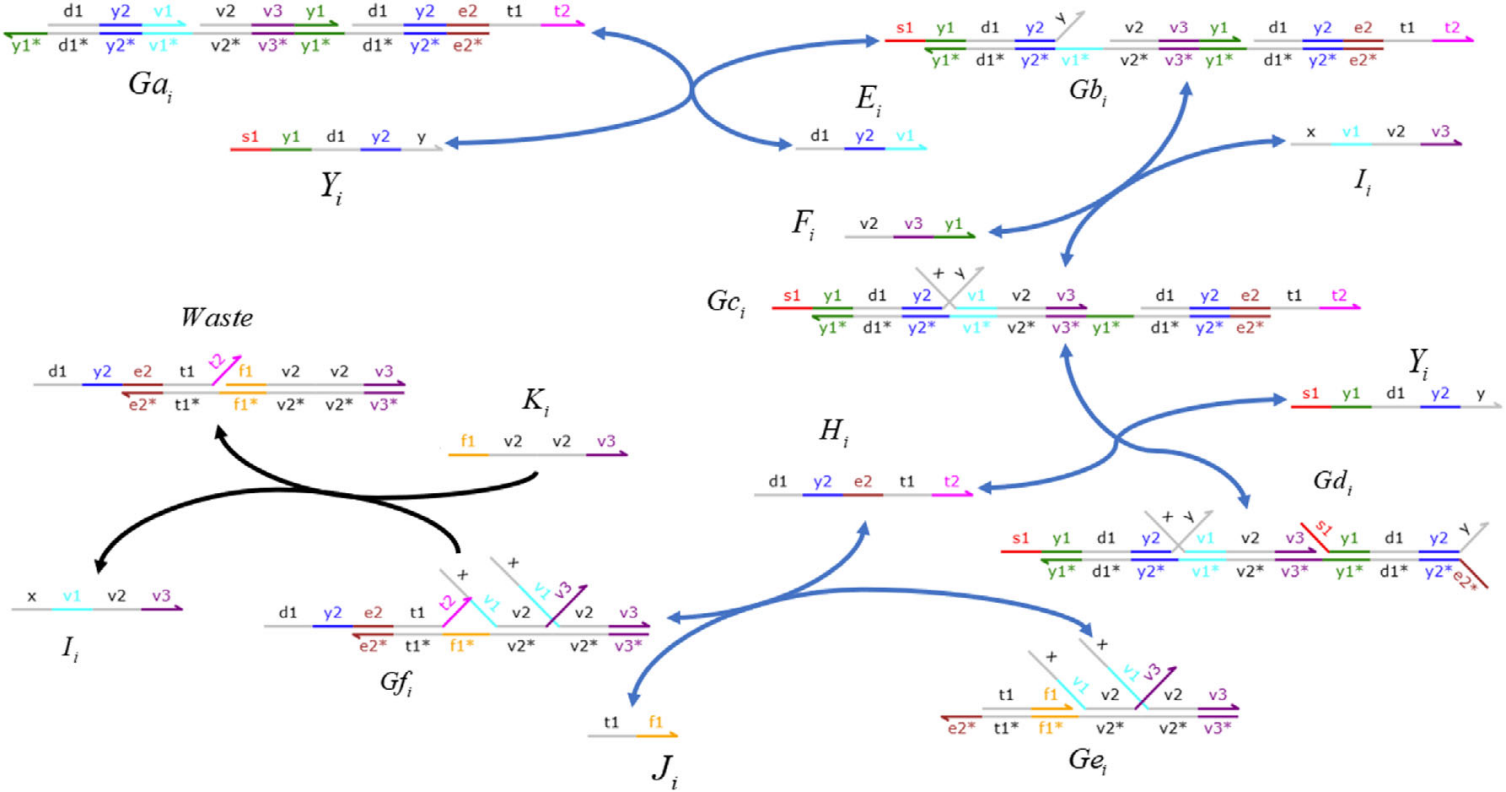
Schematic representation of DNA reactions of Equation (77).

**Catalysis reaction model 2 (Figure** **13** **)**

(77) $$2Y_{i}+I_{i}\overset{k_{i}}{\to }2I_{i}\Leftrightarrow \left\{\begin{array}{cc} & Y_{i}+Ga_{i}\underset{q_{max}}{\overset{q_{i}}{⟷}}E_{i}+Gb_{i}\\ & Gb_{i}+I_{i}\underset{q_{max}}{\overset{q_{m}}{⟷}}F_{i}+Gc_{i}\\ & Gc_{i}+Y_{i}\underset{q_{max}}{\overset{q_{m}}{⟷}}H_{i}+Gd_{i}\\ & H_{i}+Ge_{i}\underset{q_{max}}{\overset{q_{m}}{⟷}}J_{i}+Gf_{i}\\ & Gf_{i}+K_{i}\overset{q_{max}}{\to }2I_{i}+Waste \end{array}\right.,q_{i}=k_{i}$$

**Figure 14:**
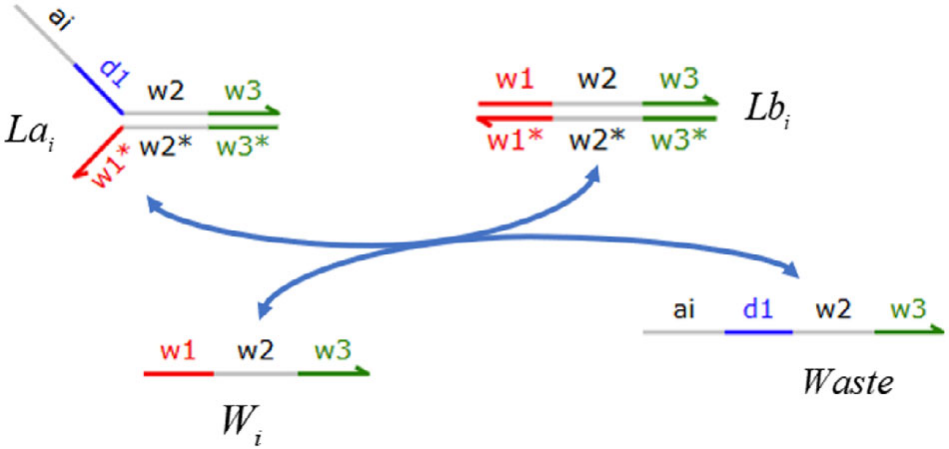
Schematic representation of DNA reactions of Equation (78).

**Adjustment reaction model (Figure** **14** **)**

(78) $$W_{i}+La_{i}\underset{kb_{i}}{\overset{ka_{i}}{⟷}}Lb_{i}\Leftrightarrow \left\{W_{i}+La_{i}\underset{qb_{i}}{\overset{qa_{i}}{⟷}}Lb_{i},\right.qa_{i}=ka_{i},qb_{i}=kb_{i}$$

### 3.2 DNA Implementation of Partial Derivative Calculation

#### 3.2.1 Calculation of Partial Derivatives of Single Variable Terms

**Figure 15:**
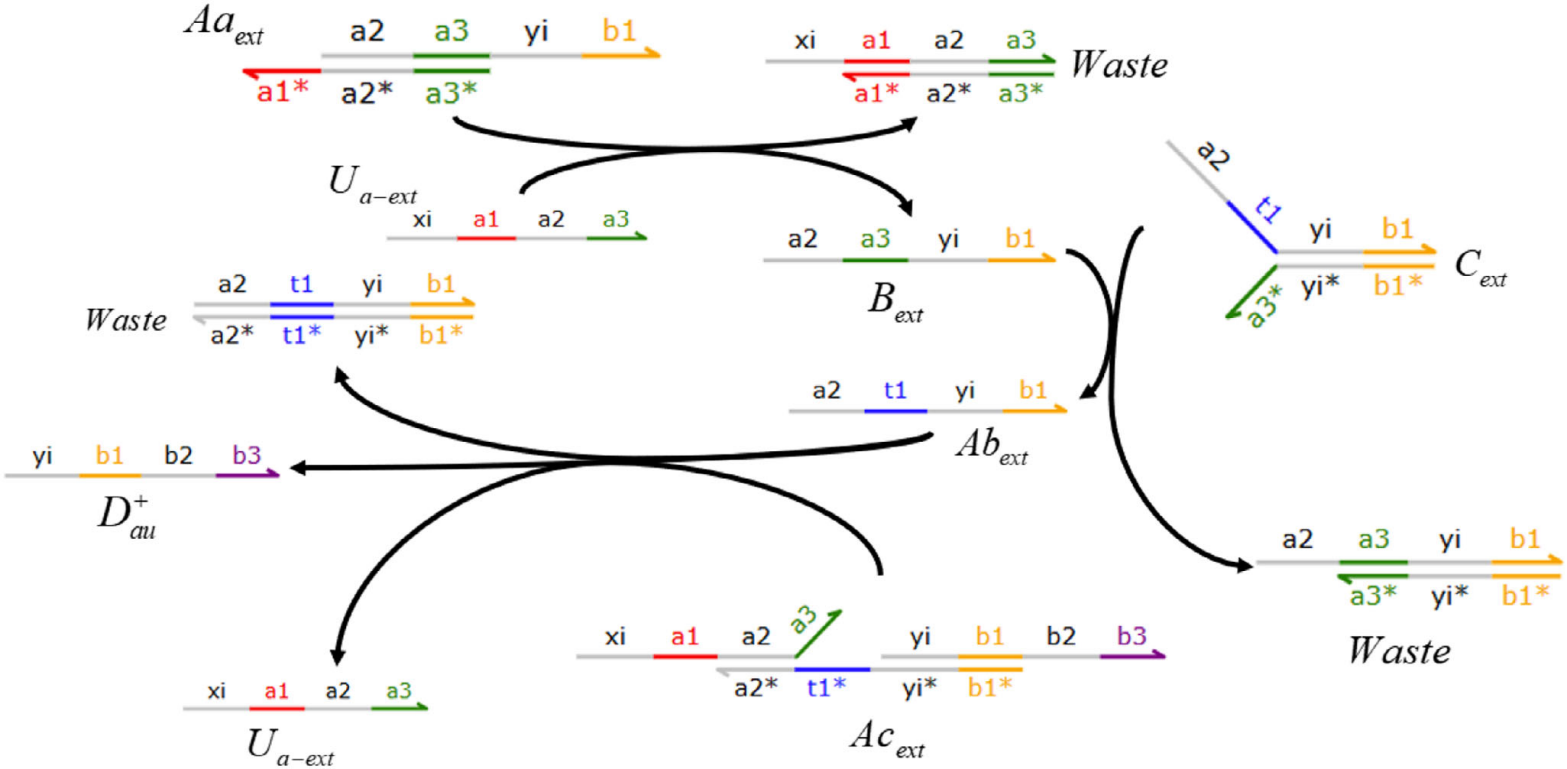
Schematic representation of DNA reactions of Equation (79).

**Catalysis reaction model (Figure** **15** **)**

(79) $$\begin{array}{ccc} & & U_{a-ext}\overset{k_{diff}}{\to }U_{a-ext}+D_{au}^{+}\Leftrightarrow \\ & & \times \left\{\begin{array}{cc} & U_{a-ext}+Aa_{ext}\overset{q_{i}}{\to }B_{ext}+Waste\\ & B_{ext}+C_{ext}\overset{q_{max}}{\to }Ab_{ext}+Waste\\ & Ab_{ext}+Ac_{ext}\overset{q_{max}}{\to }U_{a-ext}+D_{au}^{+}+Waste \end{array}\right.,q_{i}=\frac{k_{diff}}{C_{\max}} \end{array}$$

**Annihilation reaction module**

(80) $$U_{mm}+U_{nn}\overset{\gamma_{a}}{\to }⌀\Leftrightarrow \left\{\begin{array}{cc} & U_{mm}+Ra_{i}\underset{q_{max}}{\overset{q_{i}}{⟷}}R_{i}+Rb_{i}\\ & Rb_{i}+U_{nn}\overset{q_{max}}{\to }Waste \end{array}\right.,q_{i}=\gamma_{a}$$

**Figure 16:**
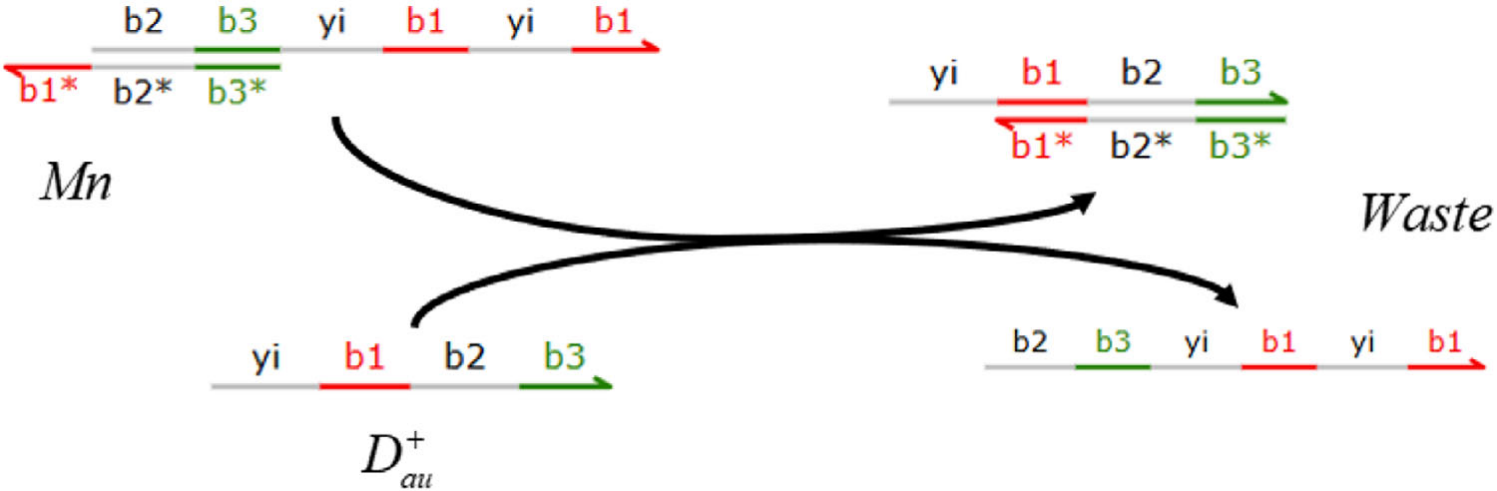
Schematic representation of DNA reactions of Equation (81).

**Degradation reaction model (Figure** **16** **)**

(81) $$D_{au}^{+}\overset{k}{\to }⌀\Leftrightarrow \left\{D_{au}^{+}+Mn\overset{q_{i}}{\to }\right.⌀,q_{i}=\frac{k}{C_{\max}}$$

#### 3.2.2 Calculation of Partial Derivatives of Compound Terms

**Catalysis reaction model**

(82) $$B\overset{k}{\to }B+E\Leftrightarrow \left\{\begin{array}{cc} & B+Aa\overset{q_{i}}{\to }B^{*}+Waste\\ & B^{*}+C^{*}\overset{q_{max}}{\to }Ab+Waste\\ & Ab+Ac\overset{q_{max}}{\to }B+E+Waste \end{array}\right.,q_{i}=\frac{k}{C_{\max}}$$

**Degradation reaction model 1**

(83) $$X+E\overset{k_{i}}{\to }E\Leftrightarrow \left\{\begin{array}{cc} & X+Oa\underset{q_{max}}{\overset{q_{i}}{⟷}}C+Ob\\ & Ob+E\underset{q_{max}}{\overset{q_{max}}{⟷}}D+Oc\\ & D+Od\overset{q_{max}}{\to }E+Waste \end{array}\right.,q_{i}=k_{i}$$

**Degradation reaction model 2**

(84) $$G\overset{k}{\to }⌀\Leftrightarrow \left\{G+M^{*}\overset{q_{i}}{\to }\right.⌀,q_{i}=\frac{k}{C_{\max}}$$

### 3.3 DNA Implementation of Partial Differential Equation Solving & Objective Function Verification

**Catalysis reaction mode 1**

(85)

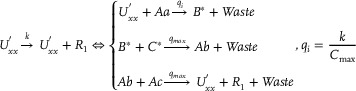




**Degradation reaction model**

(86) $$R_{1}\overset{k}{\to }⌀\Leftrightarrow \left\{R_{1}+Mn^{*}\overset{q_{i}}{\to }\right.⌀,q_{i}=\frac{k}{C_{\max}}$$

**Catalysis reaction mode 2**

(87)

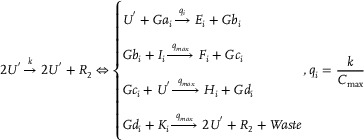




**Catalysis reaction mode 3**

(88)

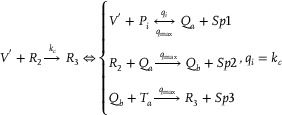




**Catalysis reaction mode 4**

(89) $$\begin{array}{ccc} & & X+T\overset{k}{\to }X+T+Cnext\Leftrightarrow \\ & & \times \left\{\begin{array}{cc} & X+A_{n}\underset{q_{max}}{\overset{q_{i}}{⟷}}Ea_{i}+Eb_{i}\\ & T+Eb_{i}\overset{q_{max}}{\to }Ec_{i}+Waste\\ & Ec_{i}+Dc_{i}\overset{q_{max}}{\to }Gc_{i}+Waste\\ & Gc_{i}+Dd_{i}\overset{q_{max}}{\to }X+T+Cnext+Waste \end{array}\right.,q_{i}=k \end{array}$$

Remark 4 For DNA realization, *Set*_*AS*_ listed in **Table** **1** represents auxiliary substances involved in the reactions, whereas *Set*_*IS*_ denotes intermediate substances. The subset *Set*_*IW*_ comprises the inert wastes that does not interact with other species engage in subsequent interactions. In addition, *C*_max _ indicates the initial concentration of auxiliary substances, while *q*_max _ denotes the maximum strand‐displacement rate. The actual reaction rate *q*_*i*_ is obtained from the corresponding DNA implementations. To mitigate the impact of auxiliary species consumption on kinetic process, the concentration of *Set*_*AS*_ in Equations (74)–(89) is initialized at its maximum value.

**Table 1:** Classification of species in the DSD reactions of Equations (74)‐(89).

Classification	Species
${\mathrm{Set}}_{\text{AS}}$	$\begin{array}{c} A_{n},Hd_{i},Oa_{i},Od_{i},Qa_{i},\theta_{n},Ga_{i},Ge_{i},\\ k_{i},Aa_{ext},C_{ext},Ac_{ext},Ra_{i},Aa,C^{*},\\ Ac,Oa,Od,P_{i},T_{a},Dc_{i},Dd_{i} \end{array}$
${\mathrm{Set}}_{\text{IS}}$	$\begin{array}{c} Hb_{i},B_{i},He_{i},Ob_{i},D_{i},Qb_{i},Gb_{i},Gc_{i},\\ H_{i},Gf_{i},B_{ext},Ab_{ext},Rb_{i},B^{*},Ab,\\ Ob,D,Qa,Qb,Eb_{i},Ec_{i},Gc_{i} \end{array}$
${\mathrm{Set}}_{\text{IW}}$	$\begin{array}{c} Ha_{i},Hc_{i},\text{Waste},C_{i},Oc_{i},E_{i},F_{i},Gd_{i},\\ J_{i},R_{i},C,Oc,Sp_{1},Sp_{2},Sp_{3},Ea_{i},⌀ \end{array}$

## 4 Results

Based on the design ideas mentioned above, the neural network scheme that utilizes augmented matrix to realize the solution of partial differential equations in biological Brussels verified by utilizing the function verification module can be implemented through the DSD mechanism. The feasibility of DSD reaction realization has been confirmed using Visual DSD software. All reaction rates and total substrate values involved in the CRNs‐based neural networks with augmented matrices, partial derivative calculations (integrating the principles of membrane diffusion and division operations) and the Brusselator partial differential equation are provided in **Tables** 2, 3, 4, with feasible values of *C*_max _ = 1000*nM* and *q*_max _ = 10^7^/*M*/*s*. The simulation results are then methodically examined, with an emphasis on three aspects: overall error *Loss*, the objective function verification module error *Error*_1_, and partial differential equation computation error *Error*_2_. A quantitative comparison of actual simulation results and theoretical values in biological PDE calculations is shown in **Table** 5.

**Table 2:** Parametric representation for augmented matrix‐based neural network response rates and weights.

Parameters	Descriptions	Nominal‐Values
*k*_1_	Input layer rate	6.0 × 10^−5^s^−1^
*k*_2_	Hidden layer rate	6.0 × 10^−5^s^−1^
*k*_3_	Output layer rate	6.0 × 10^−5^s^−1^
*k*_4_	Activation function rate	6.0 × 10^−5^s^−1^
*k*_5_, *k*_7_, *k*_9_, *k*_11_, *k*_13_	Forward reaction rate	3.65 × 10^−6^~s^−1^
*k*_6_, *k*_8_, *k*_10_, *k*_12_, *k*_14_	Reverse reaction rate	9.0 × 10^−5^s^−1^
*W*_11_	Input layer weight	0.63
*W*_12_	Input layer weight	0.24
*W*_13_	Input layer weight	0.06
*W*_14_	Input layer weight	0.42
*W*_21_	Input layer weight	0.39
*W*_22_	Input layer weight	0.36
*W*_23_	Input layer weight	0.06
*W*_24_	Input layer weight	0.26
*W*_31_	Hidden layer weight	0.11
*W*_32_	Hidden layer weight	0.42
*W*_33_	Hidden layer weight	0.09
*W*_34_	Hidden‐layer weight	0.01
*W*_41_	Hidden layer weight	0.16
*W*_42_	Hidden layer weight	0.06
*W*_43_	Hidden layer weight	0.35
*W*_44_	Hidden layer weight	0.38
*W*_51_	Output layer weight	0.19
*W*_52_	Output layer weight	0.21

**Table 3:** Parametric representation for partial derivative calculations.

Parameters	Descriptions	Nominal‐Values
*k*_diff_	Reaction rate	4.0 × 10^−4^~s^−1^
*k*	Reaction rate	3.0 × 10^−3^~s^−1^
*k*_fast_	Degradation reaction rate	10
α_*a*_	Catalytic reaction rate	5.0 × 10^−4^‐s^−1^
β_*a*_	Catalytic reaction rate	5.0 × 10^−4^‐s^−1^
δ_*a*_	Degradation reaction rate	5.0 × 10^−4^‐s^−1^
γ_*a*_	Annihilation reaction rate	1.0
*k*_*x*_	Reaction rate	6.0 × 10^−4^‐s^−1^

**Table 4:** Parametric representation for the Brusselator partial differential equation.

Parameters	Descriptions	Nominal‐Values
*D*_*u*_	Constant	0.5
*k*_*a*_	Constant	1.0
*k*_*b*_	Constant	0.3
*k*_*c*_	Constant	0.2
*k*_*d*_	Constant	0.1
*A*′	Constant	2
*B*′	Constant	2
*V*′	Constant	1

**Table 5:** Quantitative comparison of actual simulation results and theoretical values in biological PDE calculations.

Parameters	Descriptions	Actual concentration(*nM*)	Theoretical concentration(*nM*)
*Error*_1_	Neural network output error	0	0
*Error*_2_	Partial differential equation (PDE) error	0.02	0
*Loss*	Overall error	0.02	0
*P*	Neural network output	1.34	1.34
P*	Target function output	1.34	1.34
$\frac{\partial U(x,t)}{\partial x}$	First order partial derivative of variable *x*	1.86	1.85
$\frac{\partial^{2}U\left(x,t\right)}{\partial x^{2}}$	Second order partial derivative of variable *x*	2	2
$\frac{\partial U(x,t)}{\partial t}$	First order partial derivative of variable *t*	2.4	2.4
*U*_*x*_	Sum of terms of a single variable: *x*	0.64	0.64
$\frac{\partial U_{x}}{\partial x}$	First order partial derivative of variable *x*	1.6	1.6
*U*_*combt*_	Sum of composite terms of variable *x*	0.2	0.2
$\frac{\partial U_{combt}}{\partial x}$	First order partial derivative of variable *x*	0.25	0.25
*U*_*t*_	Sum of terms of a single variable *t*	0.5	0.5
$\frac{\partial U_{t}}{\partial t}$	First order partial derivative of variable *t*	2	2
$\frac{\partial U_{combt}}{\partial t}$	First order partial derivative of variable *t*	0.4	0.4

### 4.1 Overall Error *Loss*

**Figure 17:**
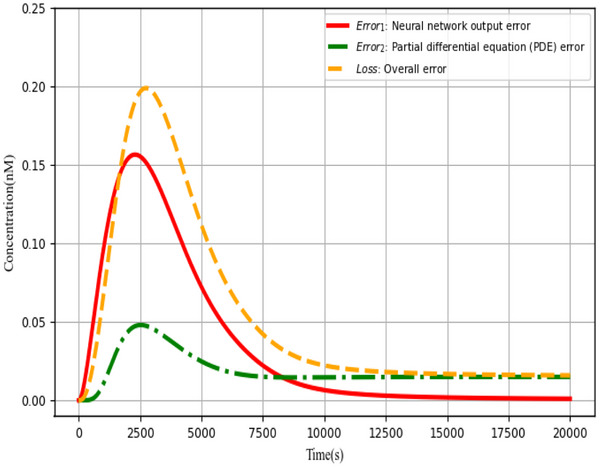
Error analysis of the augmented matrix‐based neural network for solving partial differential equations in biological Brusselator.

According to the architecture depicted in **Figure** **8**, the overall error *Loss* is an exceptionally important metric for the neural network based on the augmented matrix in solving the partial differential equations of the biological Brusselator. The overall error *Loss* consists of two parts: the error *Error*_1_ between the actual output of the DNA‐based neural network and the ideal result of the objective function; the error *Error*_2_ generated during the process of solving the partial differential equation by the neural network, and the simulation results are illustrated in **Figure** 17.

All three error curves in Figure 17 clearly follow the same trend: gradually approaching zero steadily over time. *Error*_1_ (red curve) is sufficient to control the precise output of the neural network at time *t* = 20000*s*, whereas *Error*_2_ (green curve) and *Loss* (orange curve) are stable and converge to 0.02*nM*. The approach of solving the Bio‐Brusselator partial differential equations by a neural network based on the augmented matrix is viable and effective within the acceptable error range.

### 4.2 Objective Function Verification Module Error *Error*_1_

This section focuses on the analysis of the results around the error *Error*_1_ between the augmented matrix‐based neural network output *P* and the objective function result P*, as well as the calculation of partial derivatives for each variable.

#### 4.2.1 *Error*_1_ Between The Augmented Matrix‐Based Neural Network‐Based Output *P* and The Objective Function Result P*

**Figure 18:**
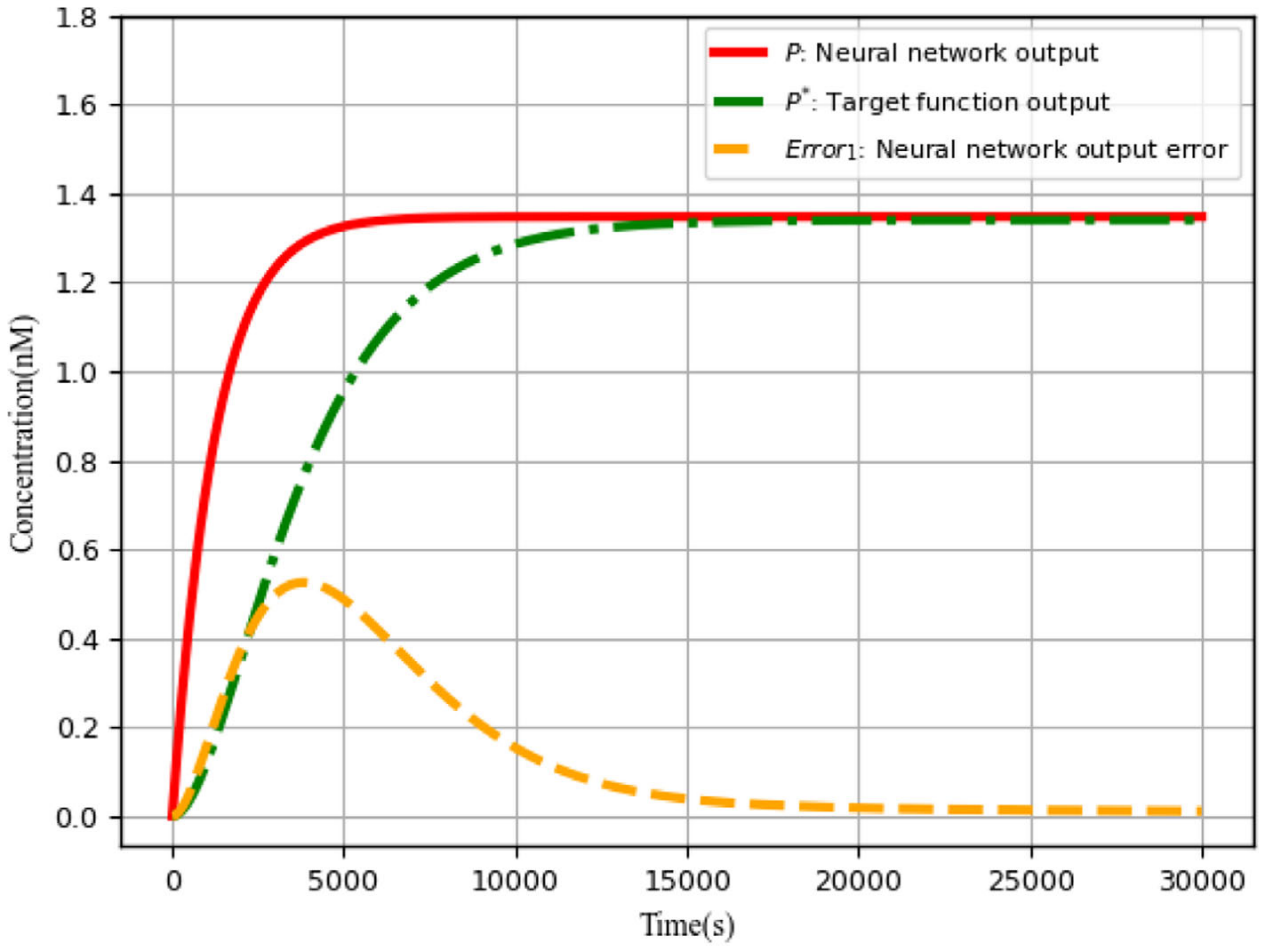
Neural network output error analysis.

The augmented matrix‐based neural network output error analysis is depicted in **Figure** **18**. In comparison to the target function output P* (green curve), the Neural network output *P* (red curve) achieves the same targeted concentration level with a faster response time and more efficiency. In addition, Neural network output error gradually approaches zero over time, indicating that the augmented matrix‐based neural network has strong tuning ability.

#### 4.2.2 Partial Derivative Calculation of Various Variables

**Figure 19:**
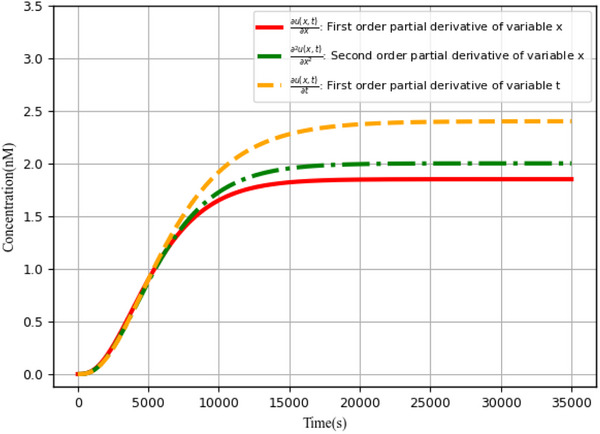
Partial derivative calculation analysis of various variable.

Partial derivative calculation of various variables is an important measure of the performance of neural networks based on augmented matrices. **Figure** **19** describes the results of calculating the first‐order and second‐order partial derivatives of the neural network outputs with respect to variables *x* and *t*, respectively (i.e., with reference to the steady‐state output concentration level).

### 4.3 Calculation Error *Error*_2_ in Partial Differential Equations

The derivation of partial derivatives of various variables, as well as the computational errors in partial differential equations, are critical for solving partial differential equations in the biological Brusselator. The first‐order and second‐order partial derivatives of the variable *x* are next examined, as well as the first‐order partial derivatives of the variable *t* and the computational error *Error*_2_ of the partial differential equation.

#### 4.3.1 Partial Derivative Calculation Analysis

**Figure 20:**
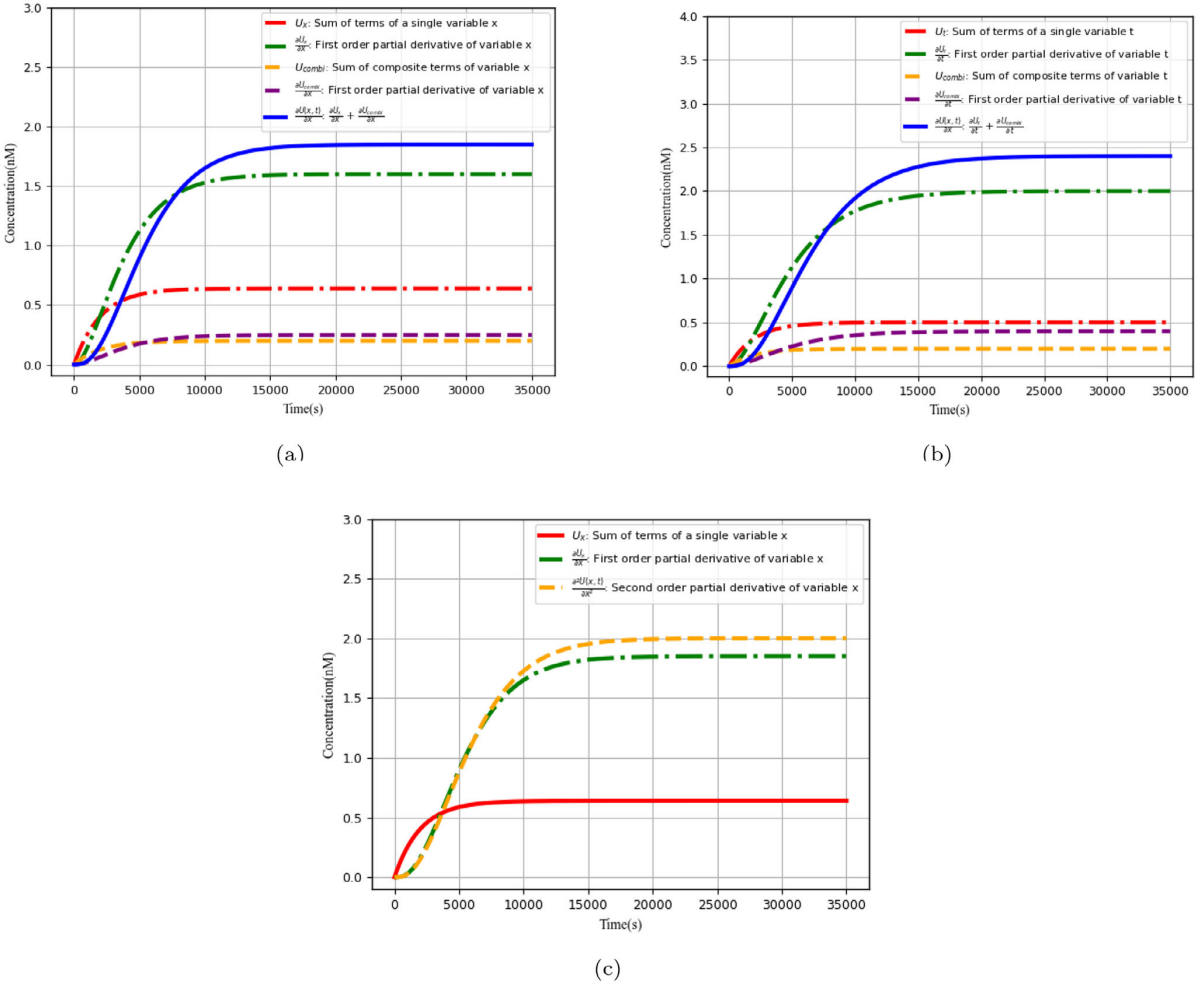
Partial derivative calculation analysis. a) First order partial derivative of variable *x*, b) First order partial derivative of variable *t*, and c) Second order partial derivative of variable *x*.

**Figure** **20**a demonstrates the first‐order partial derivative computation for variable *x*. Evidently, $\frac{\partial U(x,t)}{\partial x}$ (blue curve) is divided into two parts: $\frac{\partial U_{x}}{\partial x}$ (the term accumulation *U*_*x*_ of a single variable *x* and the first‐order partial derivatives with respect to the parameter *x*, green curve) and $\frac{\partial U_{combi}}{\partial x}$ (the composite term accumulation *U*_*combi*_ of a variable *x* and the first‐order partial derivatives with respect to the parameter *x*, purple curve).

$\frac{\partial U(x,t)}{\partial t}$ consists of two parts: $\frac{\partial U_{t}}{\partial t}$ (the term accumulation *U*_*t*_ of a single variable *t* and the first‐order partial derivatives with respect to the parameter *t*) and $\frac{\partial U_{combi}}{\partial t}$ (the composite term accumulation *U*_*combi*_ of a variable *t* and the first‐order partial derivatives with respect to the parameter *t*). The first‐order partial derivatives of the variable *t* are computed, as shown in Figure 20(b).

The second‐order partial differentiation of the variable *x* is basically a second derivative calculation utilizing the findings of the first‐order partial derivatives, and the simulation results are illustrated in Figure 20(c).

#### 4.3.2 Partial Differential Equation (PDE) Error *Error*_2_

**Figure 21:**
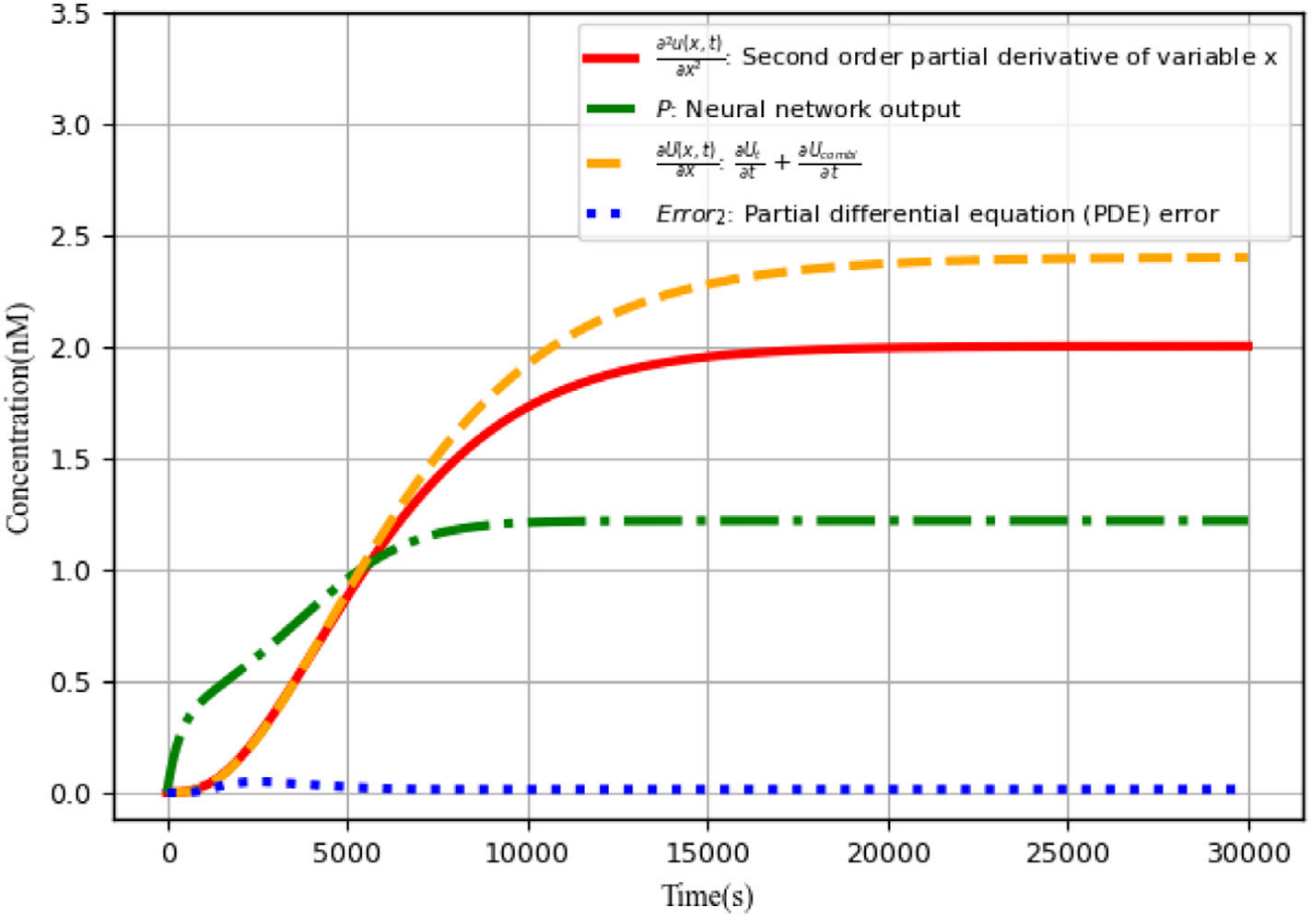
Partial differential equation (PDE) error analysis.

The augmented matrix‐based neural network scheme for solving partial differential equations in biological Brussels is feasible and effective, as demonstrated by **Figure** **21**. The error (blue curve) generated by the partial differential equation solving process gradually tends to zero over time, which is within a certain allowable error range.

## 5 Conclusion 

In this paper, the theoretical and technological development of molecular computing is advanced through a triple innovation. The augmented matrix encoding strategy transforms abstract neural network parameters into tunable DNA biochemical reaction rates and realizes multi‐parameter parallel computation using strand displacement cascade reactions; the partial derivative computation scheme based on membrane diffusion‐division synergistic mechanism fills the theoretical gap in spatial differential computation of molecular systems by simulating partial differential terms through precisely designed reaction networks; and the adaptive biochemical partial differential equations solver supporting biological partial differential equations solving DNA neural network architecture, which verifies the feasibility of continuous mathematical modeling in biochemical systems. Especially crucial is that the error feedback‐driven weight adjustment mechanism simulates the principle of biological homeostatic regulation, which provides a new idea for constructing autonomous molecular controllers. The framework not only extends DNA computation from discrete logic to continuous differential mathematics, but its molecular parallelism and continuous computing capability also enables real‐time dynamic analysis (e.g., metabolic flux redistribution simulation) of complex biological networks. This breakthrough provides new ideas for synthetic biology and opens up a new path for developing molecular‐scale artificial intelligence systems with embedded physical intelligence.

## 6 Experimental Section

Chemical reaction networks (CRNs): Complex chemical reaction networks provide a framework to capture the intricate behaviors of chemical systems, even when such systems are purely theoretical. These networks are typically modeled by differential equations based on mass action kinetics, as exemplified by the representation of system (90):

(90) $$\begin{array}{cc} & \;\text{i:}\;S_{a}+S_{b}\overset{\theta }{⟶}S_{c}\\ & \;\text{ii:}\;S_{a}\overset{k}{⟶}S_{a}+S_{b}\\ & \;\text{iii:}\;S_{b}\overset{\gamma }{⟶}⌀\\ & \;\text{iv:}\;S_{c}\overset{\mu }{⟶}2S_{c} \end{array}$$

The parameters θ and γ indicate the binding and degradation rates, and *k* denotes the catalytic rate. The differential equations of Equation (90) can be written as:

(91) $$\begin{array}{cc} & \frac{d\left[S_{a}\right]}{dt}=-\theta *S_{a}S_{b}\\ & \frac{d\left[S_{b}\right]}{dt}=-\theta *S_{a}S_{b}+k*S_{a}-\gamma *S_{b}\\ & \frac{d\left[S_{c}\right]}{dt}=\theta *S_{a}S_{b}+\mu *S_{c} \end{array}$$

In addition, the corresponding stoichiometry matrix *M*, which encapsulates the fundamental stoichiometric relationships, can be systematically derived.

(92)

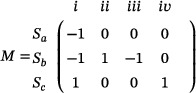

Biological signaling in biochemical circuits fundamentally relies on concentration‐based quantification, though constrained by the inherent non‐negativity of chemical species concentrations that precludes direct expression of negative values. The dual‐rail encoding strategy resolves this limitation through differential pair representation, where signal *r* is mathematically defined as *r* = *r* ^+^ − *r* ^−^ through opposing molecular species concentrations, with the corresponding kinetic relations $\dot{r}={\dot{r}}^{+}-{\dot{r}}^{-}$ maintaining mathematical consistency while allowing comprehensive signal representation.

DNA strand displacement reaction (DSD): Integrated complex chemical reactions are utilized in designing circuits capable of realizing desired behavior; to meet functional requirements, the molecules are structured as DNA strands by binding toeholds and their complementary toeholds.

For example, the DSD reactions of equation $S_{a}+S_{b}\overset{\theta }{⟶}S_{c}$ in Equation (90) are Equation (93).

(93) $$\begin{array}{cc} & S_{a}+Ra\underset{q_{\max}}{\overset{q_{i}}{⇄}}Zp1+Zp2\\ & S_{b}+Zp1\overset{q_{\max}}{⟶}Zp3+Zp4\\ & Zp4+Rb\overset{q_{\max}}{⟶}S_{c}+Zp5 \end{array}$$

**Figure 22:**
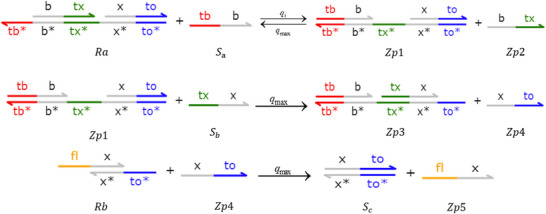
Schematic representation of DSD reactions of Equation (93).

The chemical species *Ra* and *Rb* are classified as substrates, whereas *S*_*a*_ and *S*_*b*_ are identified as reactants, with *S*_*c*_ serving as the target product. As illustrated in **Figure** **22**, the reaction mechanism comprises a combination of multiple reactions involving single‐stranded and double‐stranded DNA species. Additionally, methodologies for implementing DNA strand displacement (DSD) in broader chemical reaction networks (CRNs), including degradation pathways, are systematically detailed in Section 4.

### Simulation Methods

**Figure 23:**
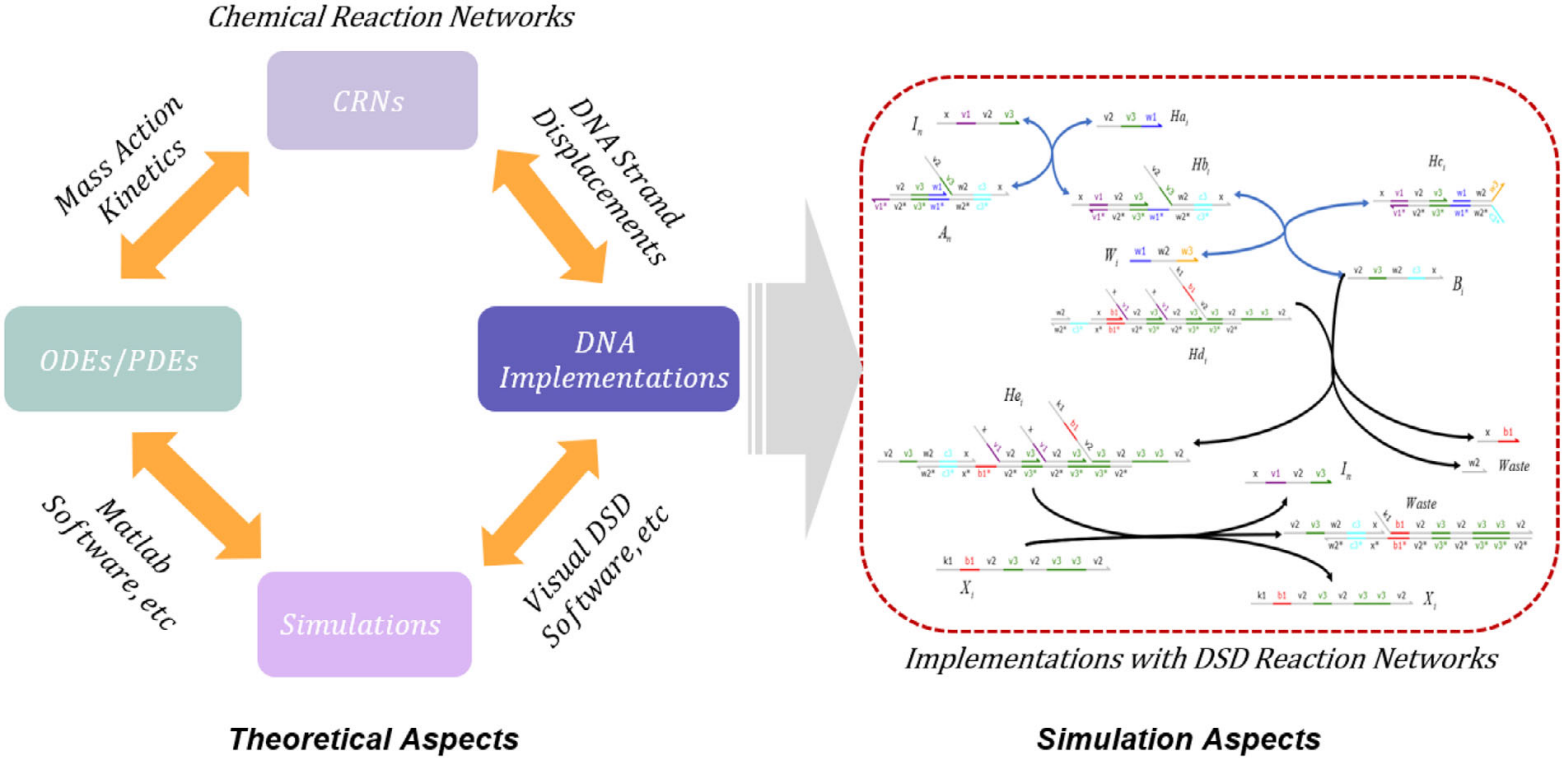
Technical roadmap for the entire research framework.

This work relies on Visual DSD software for verification, and the overall research framework adheres to the technical roadmap depicted in **Figure** **23**.

### Mathematical derivation and verification

The partial differential equation that should be solved is:

(94)

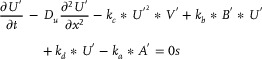

Assume the solution is a standard quadratic function:

(95) $$U\left(x,t\right)=ax^{2}+bt^{2}+cxt$$

where a, b, c > 0.

Calculation of partial derivatives of solution functions:

(96) $$\begin{array}{cc} & \frac{\partial U(x,t)}{\partial t}=\frac{\partial (ax^{2}+bt^{2}+cxt)}{\partial t}=2bt+cx\\ & \frac{\partial U(x,t)}{\partial x}=\frac{\partial (ax^{2}+bt^{2}+cxt)}{\partial x}=2ax+ct\\ & \frac{\partial U^{2}\left(x,t\right)}{\partial x^{2}}=\frac{\partial (2ax+ct)}{\partial x}=2a \end{array}$$

Let *D*_*u*_ = *m*_1_, *k*_*c*_ = *m*_2_, *V*′ = *v*_1_, *k*_*a*_ = *m*_3_, *A*′ = *v*_2_, *k*_*b*_ = *m*_4_, *B*′ = *v*_3_, *k*_*d*_ = *m*_5_; substituting Equation (96) into Equation (94), the following result can be obtained:

(97) $$\begin{array}{ccc} & & \left(2bt+cx\right)-m_{1}\left(2a\right)-m_{2}v_{1}{\left(ax^{2}+bt^{2}+cxt\right)}^{2}-m_{3}v_{2}\\ & & +m_{4}v_{3}\left(ax^{2}+bt^{2}+cxt\right)+m_{5}\left(ax^{2}+bt^{2}+cxt\right)=0 \end{array}$$

Additional sorting can be obtained:

(98) $$\begin{array}{cc} & \left(2bt+cx\right)-m_{1}\left(2a\right)-m_{2}v_{1}{\left(ax^{2}+bt^{2}+cxt\right)}^{2}\\ & \;-m_{3}v_{2}+\left(m_{4}v_{3}+m_{5}\right)\left(ax^{2}+bt^{2}+cxt\right)=0 \end{array}$$

### Steady‐state condition analysis:

In order to satisfy that *x* = 0.8, *t* = 0.5 is a steady‐state solution, the partial differential equation is required to valid. Substituting a = b into Equation (95), Equation (99) can be produced.

(99) $$\begin{array}{cc} & U\left(0.8,0.5\right)=a{\left(0.8\right)}^{2}+b{\left(0.5\right)}^{2}+c\left(0.8\right)\left(0.5\right)=0\text{.64a+0}\text{.25b+0}\text{.4c} \end{array}$$

Compute partial derivatives and other terms:

(100) $$\begin{array}{cc} & {\left.\frac{\partial U(x,t)}{\partial t}\right|}_{x=0.8,t=0.5}=2b\left(0.5\right)+c\left(0.8\right)=b+0.8c\\ & {\left.\frac{\partial^{2}U\left(x,t\right)}{\partial x^{2}}\right|}_{x=0.8,t=0.5}=2a \end{array}$$

Substituting Equations (99) and (100) into Equation (94), the following results are obtained:

(101) $$\begin{array}{cc} & \left(b+0.8c\right)-2m_{1}a-m_{2}v_{1}{\left(0.64a+0.25b+0.4c\right)}^{2}-m_{3}v_{2}\\ & \;+\left(m_{4}v_{3}+m_{5}\right)\left(0.64a+0.25b+0.4c\right)=0 \end{array}$$

**Parameter determination**:

**Constraints**

- Parameters *a*, *b*, *c*, *m*_1_, *m*_2_, *m*_3_, *m*_4_, *m*_5_, *v*_1_, *v*_2_, *v*_3_ > 0
- The equation satisfies the steady‐state solution at *x* = 0.8, *t* = 0.5.

**Parameter Design**

(102) $$\begin{array}{cc} & a=1,b=2,c=0.5,m_{1}=0.5,m_{2}=0.2,m_{3}=1,\\ & m_{4}=0.3,m_{5}=0.1,v_{1}=1,v_{2}=2,v_{3}=2 \end{array}$$

Substituting the parameters in Equation (95), we can obtain:

(103) $$U\left(x.t\right)=x^{2}+2t^{2}+0.5xt$$

Calculate the terms for *x* = 0.8, *t* = 0.5:

(104) $$\begin{array}{cc} & U\left(0.8,0.5\right)={\left(0.8\right)}^{2}+2{\left(0.5\right)}^{2}+0.5\left(0.8\right)\left(0.5\right)=1.34\\ & \frac{\partial U}{\partial t}=2bt+cx=2\left(2\right)\left(0.5\right)+0.5\left(0.8\right)=2.4\\ & \frac{\partial^{2}U}{\partial x^{2}}=2a=2 \end{array}$$

Substitute Equation (104) into the Equation (94) to verify:

(105) $$\begin{array}{c} \left(2.4\right)-2\left(0.5\right)\left(2\right)-0.2\left(1\right){\left(1.34\right)}^{2}-\left(1\right)\left(1\right)+\left(0.3\left(2\right)+0.1\right)\left(1.34\right)\\ \;=-0.02112\approx 0 \end{array}$$

Equation (103) is confirmed to be true, therefore

(106) $$U\left(x,t\right)=x^{2}+2t^{2}+0.5xt$$

is the desired steady‐state solution.

## Conflict of Interest

The authors declare no conflict of interest.

## Author contributions

Y.X. and T.S. conceived the study. Y.X. designed the methodology and prepared the original draft. T.M., A.R.‐P. and T.S. provided conceptual guidance and wrote the manuscript. J.W., P.Z. and T.S. reviewed and edited the manuscript. T.S. supervised the study and acquired funding.

## Data Availability

The data that support the findings of this study are available from the corresponding author upon reasonable request.
